# Clerodane Diterpenoids with Anti-hyperglycemic Activity from *Tinospora crispa*

**DOI:** 10.1007/s13659-016-0109-3

**Published:** 2016-10-17

**Authors:** Yuan Gao, Yan-Fen Niu, Fei Wang, Ping Hai, Fang Wang, Yin-Dong Fang, Wen-Yong Xiong, Ji-Kai Liu

**Affiliations:** 1 State Key Laboratory of Phytochemistry and Plant Resources in West China, Kunming Institute of Botany, Chinese Academy of Sciences, Kunming, 650201 Yunnan People’s Republic of China; 2 Department of Chemical Engineering, Yibin University, Yibin, 644000 People’s Republic of China; 3 Graduate University of Chinese Academy of Sciences, Beijing, 100049 People’s Republic of China; 4 BioBioPha Co., Ltd, Kunming, 650201 People’s Republic of China; 5 Yunnan University, Kunming, 650091 People’s Republic of China; 6 School of Pharmaceutical Sciences, South-Central University for Nationalities, Wuhan, 430074 China

**Keywords:** Anti-hyperglycemia

## Abstract

**Abstract:**

Four new clerodane diterpenoids, tinosporols A–C (**2**–**4**) and tinosporoside A (**5**), together with six known analogues were isolated from the vines of *Tinospora crispa*. Their structures were established by extensive spectroscopic analysis. The relative configuration at C-12 in the known diterpenoid borapetoside E (**1**), the major component of the plant, was firstly established with the aid of molecular model. Compound **1** significantly reduced serum glucose levels at dose-dependent manners in alloxan-induced hyperglycemic mice and db/db type 2 diabetic mice.

**Graphical Abstract:**

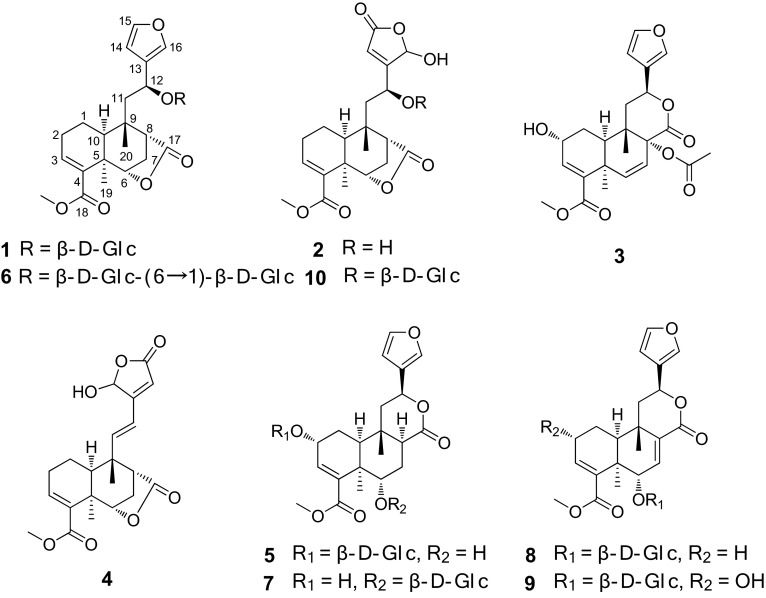

**Electronic supplementary material:**

The online version of this article (doi:10.1007/s13659-016-0109-3) contains supplementary material, which is available to authorized users.

*Tinospora crispa* (Menispermaceae) is a defoliation vine mainly distributed in Cambodia, India, Indonesia, Laos, Malaysia, Myanmar, Philippines, Thailand, and South China [1]. This plant is a prolific source of anti-hyperglycemic clerodane diterpenoids, among which borapetoside C was found to be an effective agent for the treatment of type 2 diabetes mellitus (T2DM) [2, 3].

T2DM is a chronic metabolic disorder characterized by deregulation of glucose and lipid metabolism [4]. Currently, approximately 150 million people are suffered with diabetes worldwide, and this population could increase up to 300 million by 2025. The estimated prevalence of diabetes among a representative sample of Chinese adults was 11.6 % [5]. Numerous drugs, such as rosiglitazone (ROS) and metformin (MET), have been used in the treatment of T2DM. However, treatment with synthetic drugs has been reported to lead to various side effects [6]. Therefore, searching for better agents from herbs or natural products is necessary [7]. Traditional Chinese medicines (TCMs), which have been used by the Chinese to treat illnesses for thousands of years, are combination drugs comprising several different active compounds. TCMs are better at controlling complex disease systems such as diabetes and are less prone to causing drug resistance development [8]. As part of a BioBioPha [http://www.chemlib.cn] objective to assemble a large-scale natural products library valuable in the discovery of new drug leads from TCMs [9–11], phytochemical investigation on the vines of *T. crispa* led to the isolation of four new clerodane diterpenoids, tinosporols A–C (**2–4**) and tinosporoside A (**5**), together with six known analogues. The following describes the isolation and structural elucidation of compounds **1–5**, and the anti-hyperglycemic activity of borapetoside E.

## Results and Discussion

The 95 % ethanolic extract of air-dried and powdered vines of *T. crispa* was chromatographed using silica gel, MCI CHP-20, and Sephadex LH-20 column chromatography (CC), preparative TLC, and MPLC to yield four new (**2–5**) and six known (**1**, **6**–**10**) *cis*-clerodane type diterpenoids. The known diterpenoids were identified as borapetoside E (**1**) [12], borapetoside D (**6**) [12], borapetoside B (**7**) [2], borapetoside F (**8**) [13], dehydroborapetoside B (**9**) [14], and rumphioside F (**10**), [15] respectively. Although borapetoside E (**1**), the major component of the plant, had been reported [12], the configuration at C-12 in this molecule was not determined. The relative configuration at C-12 in **1** was established spectroscopically based on computer-generated 3D drawing with minimized energy by MM2 calculation in this study.

**Fig. 1:**
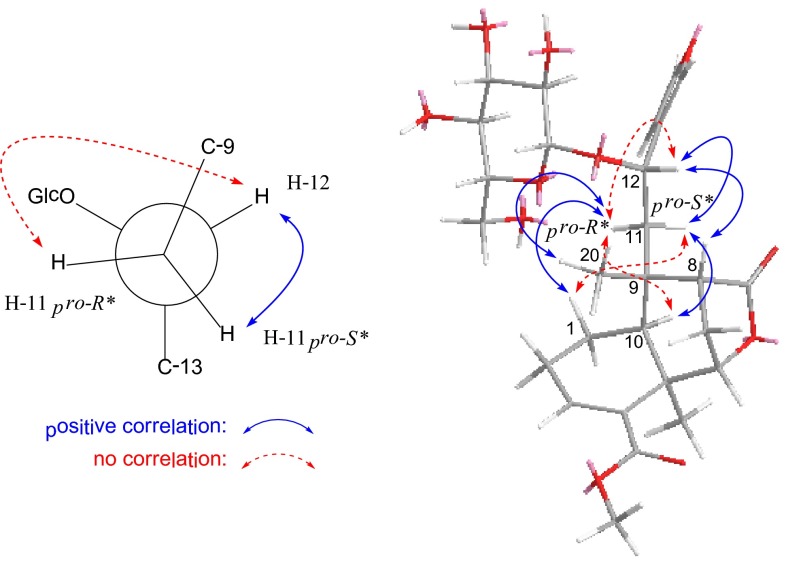
Key ROESY correlations of **1**

Compound **1** has spectral data (MS, ^1^H and ^13^C NMR) identical to those of the known compound borapetoside E [12], however, the stereochemistry at C-12 in this molecule was still undetermined. In the ^1^H NMR spectrum (Table 1), two *J* values (^3^ *J*_12,11*pro*-R*_ = 10.0 Hz; ^3^ *J*_12,11*pro*-S*_ ≈ 0 Hz) were contrasted with the coupling constants usually found in freely rotating systems (generally 5–8 Hz), indicative of that C(11)–C(12) bond bearing two large groups (butenolide and naphthane) can not rotate freely. In a Newman projection (Fig. 1), the *trans* relationship of H-11*pro*-R* and H-12 was implied by a large coupling constant (^3^ *J*_12,11*pro*-R*_ = 10.0 Hz), while the dihedral angle of H-11*pro*-S*/C-11/C-12/H-12 was supposed to be around 90°, as indicated by the ^3^ *J*_12,11*pro*-S*_ value (≈0 Hz). This conclusion was further supported by strong correlation of H-12↔H-11*pro*-S* and no correlation of H-12↔H-11*pro*-R*. In the MM2-optimized stereoview of **1** (Fig. 1), significant ROESY correlations of H-12↔H-8, H-12↔H-11*pro*-S*, and H-11*pro*-S*↔H-10 indicated that H-8, H-12, and H-11*pro*-S* are cofacial. Meanwhile, correlations of H-11*pro*-R*↔H-1 and H-11*pro*-R*↔H_3_-20, and no correlations of H-11*pro*-R*↔H-10 and H-11*pro*-S*↔H-1 revealed that H-1, 11*pro*-R*, and H_3_-20 are on the other side. Accordingly, the relative configuration at C-12 was established as S*. In a previous paper [7], it was reported that enzymatic hydrolysis of borapetoside D (**6**) yielded **1**, the relative configuration at C-12 in **6** was therefore assigned as S*.

**Table 1:** ^1^H NMR spectroscopic data for **1** and **2**

No.	**1** ^a^	**2** ^b^	**2** ^c^
1*α*	1.69, m	1.94, overlap	1.82–1.92, overlap
1*β*	1.67, m	1.94, overlap	1.82–1.92, overlap
2*α*	2.06, m	2.34/2.30, m	2.30/2.26, m
2*β*	2.16, m	2.41/2.44 td (9.4, 2.8/9.4, 3.1)	2.34, td (9.1, 3.0)/2.38, m
3	6.86, t (3.8)	7.06, t (3.8)	6.96, t (3.8)
6	5.60, d (6.1)	5.57/5.55, d (6.1)	5.28, d (6.1)
7*α*	2.03, ddd (12.5, 6.1, 5.6)	2.25/2.23, ddd (12.6, 6.4, 6.1)	2.20, ddd (12.5, 6.1, 5.1)
7*β*	1.88, d (12.5)	2.00/1.99, d (12.6)	1.84, d (12.5)
8	3.21, d (5.6)	2.59/2.61, d (6.4)	2.53, d (5.1)/2.57, m
10	1.44, dd (5.5, 4.4)	1.45, t (3.6)/1.49, dd (4.6, 2.3)	1.31/1.39, brs
11*pro*-R*	2.29, dd (14.9, 10.0)	1.94, overlap/2.06, dd (15.0, 9.1)	1.83/1.92, overlap
11*pro*-S*	1.88, brd (14.9)	1.63/1.84, d (15.1/15.0)	1.47/1.71, d (14.9/14.1)
12	5.81, brd (10.0)	4.85/4.94, d (8.4/9.1)	4.69/4.72, m
14	6.97, d (1.2)	6.07/6.06, s	5.93/6.02, s
15	7.61, t (1.6)		
16	8.20, brs	6.25, s	6.14/6.13, brs
19	1.35, s	1.34, s	1.23, s
20	1.34, s	1.26/1.27, s	1.17/1.18, s
OMe	3.66, s	3.74/3.73, s	3.67, s
*CH*_*3*_CO			
12-OH			5.37/5.30, d (5.4/5.9)
16-OH			7.89/7.86, brs
1′	4.96, d (7.8)		
2′	4.08, t (8.2)		
3′	4.16, t (8.7)		
4′	4.19, t (8.9)		
5′	3.83, m		
6′	4.30, dd (11.5, 5.7) 4.52, dd (11.5, 2.4)		

^a^Measured in pyridine-*d*_5_ (*δ*_H_ 8.71 ppm)

^b^Measured in CDCl_3_ (*δ*_H_ 7.26 ppm)

^c^Measured in DMSO-*d*_6_ (*δ*_H_ 2.49 ppm)

**Fig. 2:**
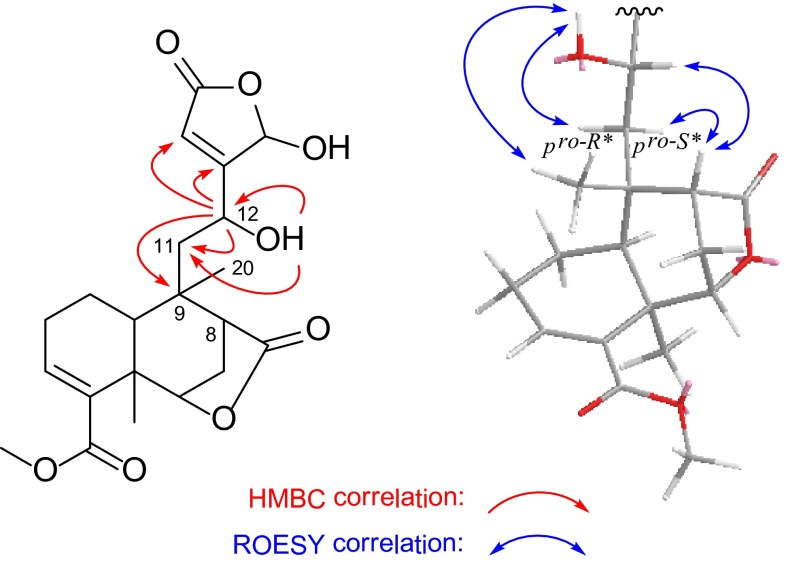
Key HMBC and ROESY correlations of **2**

Compound **2** was obtained as an inseparable C-16 epimeric mixture (3:1) where some of the signals appeared as duplicate in the NMR spectra (Tables 1, 2). Its molecular formula was determined as C_21_H_26_O_8_ from HREIMS at *m*/*z* 406.1623 [M]^+^ (calcd 406.1628), requiring 9 degrees of unsaturation. The IR spectrum suggested the presence of hydroxy (3411 cm^−1^) and carbonyl (1710 cm^−1^) functionalities. Comparison of its ^13^C NMR data with that of rumphioside F (**10**) [7] revealed a remarkable resemblance except for the absence of a set of glucopyranosyl resonances and the signal assigned to C-12 being upfield (Δ = –6.1 ppm), which suggested that **2** was an aglycone of rumphioside F. Heteronuclear multiple bond connectivity (HMBC) correlations (Fig. 1) from 12-OH to C-11/C-12 and from H-12 to C-9/C-11/C-13/C-14 further verified the location of this hydroxy group. The configurations at C-5, C-6, C-8, and C-9 were consistent with those of rumphioside F (**10**) according to a careful analysis of the ROESY spectrum. Furthermore, the stereochemistry for C-12 was established by analysis of the proton coupling constants and ROESY data (Fig. 2). The *anti* relationship of H-11*pro*-R* and H-12 and the dihedral angle of H-11*pro*-S*/C-11/C-12/H-12 (ca.90°) is reasonable by two coupling constants (^3^ *J*_12,11*pro*-R*_ = 8.4/9.1 Hz; ^3^ *J*_12,11*pro*-S*_ ≈ 0 Hz) recorded in CDCl_3_ (Table 1). In the ROESY spectrum (Fig. 1) measured in DMSO-*d*_*6*_, significant correlations of H-12↔H-8 and H-11*pro*-S*↔H-8 indicated that H-12, H-8, and H-11*pro*-S* are spatially close to each other. Similarly, correlations of 12-OH↔Me-20 and 12-OH↔ H-11*pro*-R* implied that these protons are cofacial. The relative configurations of other structural parts of **2** are identical with those of rumphioside F (**10**) [7], based on detailed analysis of ROESY spectrum and coupling constants. Hence, the structure of **2** were characterized and given the trivial name tinosporol A.

**Table 2:** ^13^C NMR spectroscopic data for **1** and **2**

No.	**1** ^a^	**2** ^b^	**2** ^c^
1	16.6, CH_2_	16.4, CH_2_	16.3, CH_2_
2	24.3, CH_2_	24.0, CH_2_	24.1, CH_2_
3	142.7, CH	142.8, CH	143.1, CH
4	134.4, C	133.8, C	133.9, C
5	39.5, C	39.1, C	39.1, C
6	82.8, CH	83.9, CH	82.6, CH
7	29.6, CH_2_	29.4, CH_2_	29.2, CH_2_
8	46.8, CH	47.7/47.4, CH	47.1/46.8, CH
9	39.8, C	39.4, C	39.4, C
10	45.8, CH	44.5/45.0, CH	45.0/45.6, CH
11	47.7, CH_2_	44.1/44.6, CH_2_	44.9/44.1, CH_2_
12	69.5, CH	64.6, CH	64.1/63.5, CH
13	128.6, C	173.5/171.0, C	175.8/173.6, C
14	110.5, CH	116.8/117.9, CH	115.9/116.7, CH
15	143.7, CH	171.0, C	171.1, C
16	140.8, CH	97.2/98.1, CH	98.7/98.0, CH
17	178.3, C	179.7, C	177.7, C
18	166.9, C	166.6, C	166.8, C
19	27.2, CH_3_	27.0, CH_3_	27.3, CH_3_
20	21.6, CH_3_	20.7/21.5, CH_3_	21.0/21.9, CH_3_
OMe	51.6, CH_3_	51.8, CH_3_	52.1, CH_3_
CH_*3*_CO			
CH_3_ *C*O			
1′	101.7, CH		
2′	75.6, CH		
3′	78.6, CH		
4′	72.2, CH		
5′	78.2, CH		
6′	63.1, CH_2_		

^a^Measured in pyridine-*d*_5_ (*δ*_C_ 149.9 ppm)

^b^Measured in CDCl_3_ (*δ*_C_ 77.0 ppm)

^c^Measured in DMSO-*d*_6_ (*δ*_C_ 39.5 ppm)

Compound **3** gave a molecular formula of C_23_H_26_O_8_ by HREIMS ([M]^+^ *m/z* 430.1634, calcd 430.1628). The ^1^H and ^13^C NMR data (Tables 3, 4) were similar to those of tinocrispol A [2] except for an additional acetyl group (*δ*_H_ 2.12; *δ*_C_ 21.1, 168.7). This group was attached at C-8 (*δ*_C_ 78.1) due to the chemical shift of C-8 in **3** showing a downfield shift (Δ = 4.8 ppm) relative to that of the corresponding hydroxyl-bearing carbon in tinocrispol A, which was also confirmed by the lack of esterification shift [2] of H-2 in **3** and weak HMBC correlation (^4^ *J*) from the acetyl proton (*δ*_H_ 2.12) to C-8. The acetyl group is *α*-oriented as indicated by a clear ROESY correlation between H-12 and acetyl proton. Consequently, the structure of compound **3** was identified as shown, and it was named tinosporol B.

**Table 3:** ^1^H NMR spectroscopic data for **3**–**5**

No.	**3** ^a^	**4** ^a^	**5** ^b^
1*α*	2.01, ddd (14.7, 10.5, 6.4)	1.94, m	2.18, ddd (14.1, 8.1, 6.8)
1*β*	2.29, brdd (14.7, 7.3)	1.64, m	2.33, dd (14.1, 8.7)
2*α*	4.56, ddd (10.5, 7.3, 2.6)	2.31–2.37, overlap	4.69, td (8.4, 4.0)
2*β*		2.31–2.37, overlap	
3	6.72, brs	7.05, t (3.7)	6.45, d (4.0)
6	6.58, d (10.4)	5.56, t (5.8)	4.45, brd (2.4)
7*α*	6.55, d (10.4)	2.22–2.27, m	1.91, ddd (14.1, 4.0, 2.4)
7*β*		2.02/2.02, d (12.6/11.9)	1.55, ddd (14.1, 12.1, 1.7)
8		2.23, overlap/2.28, d (5.3)	3.35, overlap
10	2.52, dd (6.4, 1.7)	1.84, t (6.1)	2.21, d (6.8)
11*pro*-R*	1.97, dd (13.3, 8.7)	6.50/6.53, d (16.4)	2.00, dd (14.1, 11.6)
11*pro*-S*	2.31, dd (13.3, 8.0)		2.12, dd (14.1, 6.1)
12	5.59, dd (8.7, 8.0)	6.36/6.37, d (16.4)	5.49, dd (11.4, 5.9)
14	6.41, d (1.2)	5.92/5.93, s	6.51, brd (1.2)
15	7.43, t (1.4)		7.51, t (1.7)
16	7.47, brs	6.31/6.26, s	7.59, brs
19	1.55, s	1.37, s	1.47, s
20	0.95, s	1.29, s	0.97, s
OMe	3.73, s	3.74, s	3.74, s
CH_*3*_CO	2.12, s		
12-OH			
16-OH			
1′			4.44, d (7.9)
2′			3.14, dd (9.1, 7.9)
3′			3.34, t (8.9)
4′			3.26, t (9.0)
5′			3.29, m
6′			3.65, dd (11.8, 5.8) 3.87, dd (11.8, 1.9)

^a^Measured in CDCl_3_ (*δ*_H_ 7.26 ppm)

^b^Measured in methanol-*d*_4_ (*δ*_H_ 3.30 ppm)

**Table 4:** ^13^C NMR spectroscopic data for **3**–**5**

No.	**3** ^a^	**4** ^a^	**5** ^b^
1	28.2, CH_2_	16.9, CH_2_	26.7, CH_2_
2	64.4, CH	23.9, CH_2_	72.6, CH
3	139.6, CH	142.79/142.66, CH	138.5, CH
4	136.5, C	133.78/133.81, C	141.8, C
5	37.3, C	38.6, C	41.9, C
6	138.0, CH	83.30/83.18, CH	69.3, CH
7	120.8, CH	29.83/29.81, CH_2_	28.5, CH_2_
8	78.1, C	50.86/50.71, CH	41.3, CH
9	39.2, C	42.47/42.42, C	38.3, C
10	45.3, CH	41.31,41.40, CH	51.0, CH
11	39.4, CH_2_	148.96/148.77, CH	46.2, CH_2_
12	70.9, CH	119.72/119.85, CH	72.0, CH
13	125.7, C	160.66/160.63, C	126.0, C
14	108.3, CH	117.28/117.26, CH	109.9, CH
15	144.0, CH	170.92/170.87, C	145.0, CH
16	139.4, CH	97.54/97.46, CH	141.4, CH
17	167.3, C	177.61/177.56, C	178.3, C
18	166.6, C	166.5, C	169.9, C
19	30.3, CH_3_	26.9, CH_3_	29.7, CH_3_
20	23.1, CH_3_	19.15/19.12, CH_3_	23.5, CH_3_
OMe	51.9, CH_3_	51.8, CH_3_	52.4, CH_3_
CH_*3*_CO	21.1, CH_3_		
CH_3_ *C*O	168.7, C		
1′			103.6, CH
2′			75.0, CH
3′			78.0, CH
4′			71.6, CH
5′			77.9, CH
6′			62.7, CH_2_

^a^Measured in CDCl_3_ (*δ*_C_ 77.0 ppm)

^b^Measured in methanol-*d*_4_ (*δ*_C_ 49.0 ppm)

Compound **4** was isolated as an inseparable C-16 epimeric mixture (5:4). It gave a molecular formula of C_21_H_24_O_7_ by HREIMS ([M]^+^ *m/z* 388.1521, calcd 388.1522). The ^1^H and ^13^C NMR data (Tables 3, 4) revealed that the compound closely resembled **2**. The only difference was that signals for a methylene (C-11) and a methine (C-12) in **2** were replaced by a double bond (*δ*_H_ 6.50/6.53, 6.36/6.37; *δ*_C_ 148.96/148.77, 119.72/119.85) in **4** on the basis of HMBC correlations from H-11 to C-8, C-10, C-12, C-13, and C-20. The *E*-geometry of C-11/C-12 olefin was supported by the large coupling constant (*J*_11,12_ = 16.4 Hz) and ROESY correlations of H-11/H-16 and H-12/H_3_-20. Therefore, the structure of compound **4** was determined and named tinosporol C.

Compound **5** had the molecular formula C_27_H_36_O_12_, deducing from the HREIMS ([M]^+^ *m/z* 552.2225, calcd 552.2207). The ^1^H NMR and ^13^C NMR spectra (Tables 3, 4) displayed signals very similar to those of **7 [** 2]. The principal difference between them was the location of the glucopyranosyl moiety. This moiety was linked to C-2 as indicated by HMBC correlations from the anomeric proton (*δ*_H_ 4.44) to C-2 (*δ*_C_ 72.6). The relative configuration of **5** was determined to be the same as that of **7**, based on detailed analysis of ROESY spectrum and coupling constants. Thus, the structure of **2** were established and given the trivial name tinosporoside A.

**Fig. 3:**
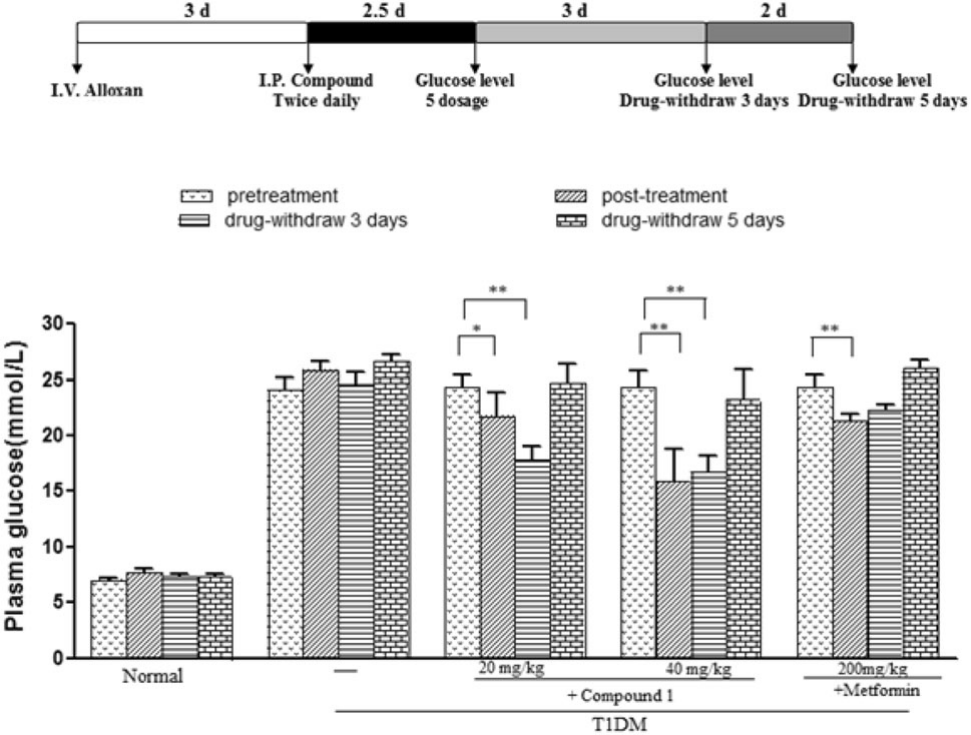
Effect of compound **1** on alloxan-induced hyperglycemia mice. Experimental procedure was shown above. Data are shown as mean ± SEM (n = 10 in each group). *P < 0.05, **P < 0.01 (Dunnett’s test). Metformin served as a positive control

Based on the usage of *T. crispa* in TMCs and corrected structure characteristics of the compound **1**, then the anti-hyperglycemia activities of compound **1** on hyperglycemic mice in vivo were evaluated. Following intravenously administrated compound **1** in alloxan-induced hyperglycemic mice which is a fast and classic type 1 diabetes mellitus (T1DM) model in pharmacological study [16]. In our study, alloxan injections markedly increased the serum glucose level from 7.0 ± 0.5 mM/L to 24.3 ± 3.7 mM/L (Fig. 3). Under this condition, compound **1** (dose of 20 mg/kg) was able to induce a significant decrease of blood glucose down to 21.7 ± 6.7 mM/L after 5 times administration (25.9 ± 2.5 mM/L before compound treatment), whereas dose of 40 mg/kg of the compound caused a further down of glucose level to 15.9 ± 9.2 mM/L, indicating a tendency of dose-dependent effect of the compound on lowing blood glucose in this animal model.

Furthermore, regarding that it is a valuable approach to extensively evaluate the drug effect on animals by evaluating its effect after drug withdrawing. Next we measured blood glucose levels on 3 or 5 days later after withdraw of compound **1**. Surprisingly, the blood glucose level was even further down to 17.9 ± 3.8 mM/L after 3-days withdrawing for the group of 20 mg/kg treatment (compared to 21.7 ± 6.7 mM/L before drug withdrawing, Fig. 3), whereas glucose level after 3-days withdrawing in group of 40 mg/kg treatment maintained at 16.7 ± 4.7 mM/L which was similar to the level before drug withdraw (15.9 ± 9.2 mM/L, Fig. 3). Explaining for these results could be the maxim of glucose being recovered by the drug could be 16 mM/L or so, so that 40 mg/kg treatment already achieved the maxim level in this approach. After withdrawing drug for 5 days, both glucose levels for groups of 20 or 40 mg/kg treatment fully recovered to the levels before drug treatments.

**Fig. 4:**
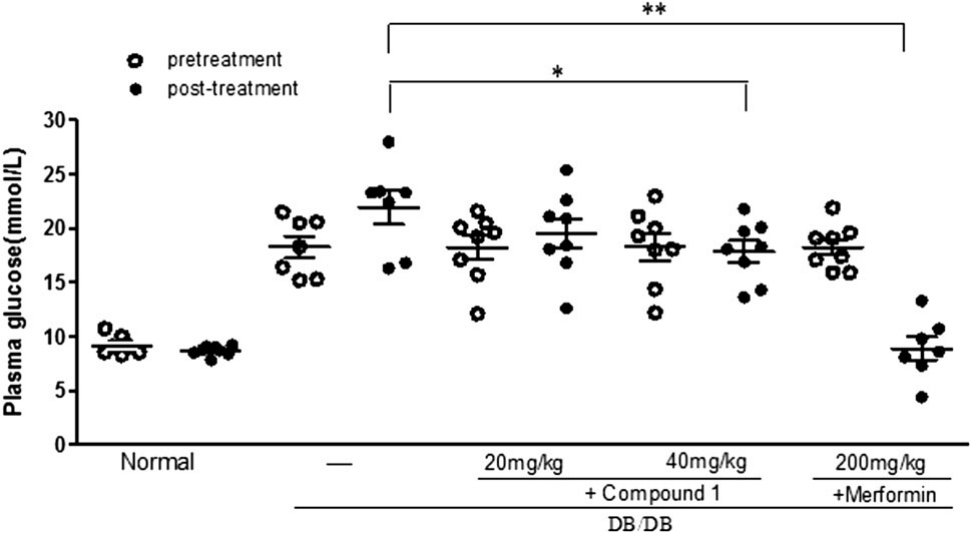
Effects of compound **1** on glucose levels of db/db mice. Data are presented as mean ± SEM (n = 10 in each group). *P < 0.05, **P < 0.01 (Dunnett’s test). Metformin served as a positive control

Furthermore, we asked that whether the effect of compound **1** on lowing blood glucose is specific for alloxan-induced hyperglycemic mice. Therefore we estimated the effect of the compound on db/db mice that is genetic animal model of which has been widely used in T2DM study. Compound **1** administration for 5 times slightly decreased glucose levels to 17.85 ± 2.83 mM/L of these mice at the dosage of 40 mg/kg (*p* = 0.041, compared to 21.93 ± 4.10 mM/L of model mice), whereas a lower dose (20 mg/kg) didn’t affect the glucose level (19.49 ± 3.91 mM/L, Fig. 4). Comparing with the strikingly decreased glucose (8.89 ± 2.80 mM/L) with MET administration, we concluded that T1DM mice are more sensitive than T2DM mice to compound **1**.

## Experimental Section

### General Experimental Procedures

Optical rotations were measured on a Jasco P-1020 automatic digital polarimeter. UV spectra were obtained in an HPLC (Agilent 1200, DAD). IR spectra were obtained using a Bruker Tensor 27 FT-IR spectrometer with KBr pellets. NMR spectra were acquired with a Bruker Avance III 600 instrument at room temperature. EIMS (including HREIMS) was measured on a Waters AutoSpec Premier P776 spectrometer. Silica gel (200–300 mesh, Qingdao Marine Chemical, Inc., Qingdao, P. R. China), MCI CHP-20 (70–150 *μ*m, Mitsubishi Chemical Corporation, Japan) and Sephadex LH-20 (Amersham Biosciences, Sweden) were used for column chromatography (CC). Medium pressure liquid chromatography (MPLC) was performed on a Büchi Sepacore System equipping with pump manager C-615, pump modules C-605, and fraction collector C-660 (Büchi Labor technik AG, Switzerland), and columns packed with Chromatorex C-18 (40–75 *μ*m, Fuji Silysia Chemical Ltd., Japan). Fractions were monitored by TLC and HPLC (Agilent 1200, Extend-C18 column, 5 *μ*m, 4.6 × 150 mm).

### Plant Material

The vines of *T. crispa* were collected in April 2012 in Simao, Yunnan Province, China. The sample was identified by Mr. Yu Chen of Kunming Institute of Botany, Chinese Academy of Sciences. The voucher specimen (No. BBP20120452) was deposited at BioBioPha Co., Ltd.

### Extraction and Isolation

The air-dried and powdered vines of *T. crispa* (15 kg) were extracted with EtOH-H_2_O (95:5, v/v; 3 × 25 L, each 5 days) at room temperature, and the solvent was removed under reduced pressure to give crude extract (ca. 1100 g), which was fractionated by silica gel CC successively eluted with petroleum ether (PE)/acetone (99:1→0:100 gradient) to give seven fractions A–G.

Fraction C (11 g) was subjected to MPLC using a stepwise gradient of MeOH/H_2_O (0→100 %) to give fractions C1–C4. Fraction C2 was separated by MPLC (MeOH/H_2_O, 45 %), followed by prep. TLC (CHCl_3_/MeOH, 20:1) to obtain **2** (27 mg). After repeated silica gel CC (CHCl_3_/MeOH, 50:1), fraction C3 afforded the precipitate **3** (30 mg).

Fraction E (180 g) was separated by silica gel CC (CHCl_3_/MeOH, 40:1→5:1) into fractions E1–E3. Fraction E1 was chromatographed over a Sephadex LH-20 column (MeOH) repeatedly to yield **1** (5.3 g) and **8** (331 mg), respectively. Fraction E2 was subjected to MCI (MeOH/H_2_O, 45→50 %) and then Sephadex LH-20 (MeOH) CC to give **5** (4 mg) and **10** (46 mg), respectively. The latter was unstable, and after the 1D NMR experiment followed by freeze drying, converted to a product less polar than **10**, which afforded **4** (18 mg) after silica gel CC (CHCl_3_/MeOH, 20:1). Compounds **7** (2.3 g) and **9** (98 mg) were obtained from fraction E3 by chromatography on a silica gel column (CHCl_3_/MeOH, 9:1).

Fraction F (90 g) was separated by silica gel CC (CHCl_3_/MeOH, 5:1→3:1) into fractions F1 and F2. Compound **6** (162 mg) was obtained from F2 by MCI CC (MeOH/H_2_O, 5→25 %).

#### Tinosporol A (**2**)

White, amorphous powder; [α]_D_^28^ −113.4 (*c* 0.05, CHCl_3_); UV (MeOH) *λ*_max_: 213 nm; IR (KBr) *ν*_max_ 3441, 2951, 1762, 1710, 1634, 1437, 1352, 1270, 1242, 1154, 1104, 1074, 952, 906 cm^−1^; ^1^H and ^13^C NMR data, see Tables 1 and 2; EI-MS: *m/z*406 [M]^+^ (12), 388 (13), 391 (62), 328 (13), 278 (28), 263 (28), 235 (100), 203 (90), 185 (22), 152 (18), 93 (29), 91 (32); HR-EI-MS: *m/z*406.1623 (calcd for C_21_H_26_O_8_, 406.1628).

#### Tinosporol B (**3**)

White, amorphous powder; [α]_D_^27^ −147.2 (*c* 0.05, CHCl_3_); UV (MeOH) *λ*_max_: 213 nm; IR (KBr) *ν*_max_ 3442, 2952, 1758, 1715, 1634, 1437, 1371, 1232, 1156, 1106, 1072, 1024, 1007, 876, 780 cm^−1^; ^1^H and ^13^C NMR data, see Tables 3 and 4; EI-MS: *m/z* 430 [M]^+^ (10), 399 (10), 370 (28), 342 (30), 294 (100), 262 (41), 203 (52), 173 (37), 122 (72), 95 (70), 91 (59); HR-EI-MS: *m/z* 430.1634 (calcd for C_23_H_26_O_8_, 430.1628).

#### Tinosporol C (**4**)

White, amorphous powder; [α]_D_^28^ −133.6 (*c* 0.05, CHCl_3_); UV (MeOH) *λ*_max_: 208, 262 nm; IR (KBr) *ν*_max_ 3432, 2949, 1760, 1710, 1641, 1452, 1436, 1351, 1268, 1243, 1228, 1181, 1160, 1223, 1101, 975, 953 cm^−1^; ^1^H and ^13^C NMR data, see Tables 3 and 4; EI-MS: *m/z* 388 [M]^+^ (75), 370 (30), 357 (33), 338 (48), 181 (50), 153 (100), 149 (61), 105 (49), 93 (83), 91 (75), 83 (60), 77 (61); HR-EI-MS: *m/z* 388.1521 (calcd for C_21_H_24_O_7_, 388.1522).

#### Tinosporoside A (**5**)

White, amorphous powder; [α]_D_^26^ −78.7 (*c* 0.05, CHCl_3_); UV (MeOH) *λ*_max_:214 nm; IR (KBr) *ν*_max_ 3442, 2925, 1720, 1632, 1440, 1383, 1252, 1158, 1101, 1073, 1026, 602 cm^−1^; ^1^H and ^13^C NMR data, see Tables 3 and 4; EI-MS: *m/z* 552 [M]^+^ (7), 390 (13), 372 (15), 309 (40), 284 (29), 119 (48), 91 (68), 69 (100); HR-EI-MS: *m/z* 552.2225 (calcd for C_27_H_36_O_12_, 552.2207).

### Anti-hyperglycemia Activity

Male ICR and db/db mice were obtained from Beijing HFK Bioscience CO., LTD (Certificate No. SCXK 2014-0004) or Nanjing Biomedical Research Institute of Nanjing University (Certificate No. SCXK 2010-0001) respectively. These animals were housed in a temperature and humidity-controlled room with a 12-hour light/dark cycle. Animals were given ad libitum access to food and water throughout the study. All of the procedures were performed in accordance with the Institute Ethical Committee for Experimental Animal Use. For inducing hyperglycemic mice, 8-week-old ICR mice were intravenously injection with freshly prepared alloxan (60 mg/kg). Serum glucose level was measured with glucose kit (Changsha Sinocare Inc., China). The mice with plasma glucose level greater than 11.1 mM/L were considered diabetic. Animals were treated with the compound **1** or MET twice daily for 2.5 days at the doses 20, 40 or 200 mg/kg as indicated in the figures.

### Statistical Analysis

The data were presented as mean ± SEM, subjected to one-way analysis of variance followed by Dunnett’s multiple comparison tests using GraphPad Prism version 5.0 software. *P < 0.05, **P < 0.01.

## Electronic Supplementary Material

Below is the link to the electronic supplementary material.

Supplementary material 1 (DOCX 3118 kb)
