# Supplementary material for: Clerodane Diterpenoids with Anti-hyperglycemic Activity from Tinospora crispa
Source: Nat Prod Bioprospect. 2016 Oct 17;6(5):247–55. doi: 10.1007/s13659-016-0109-3 (PMC5080211; doi:10.1007/s13659-016-0109-3)

**Supporting Information**

**Clerodane Diterpenoids with Anti-hypoglycemic Activity from *Tinospora crispa***

Yuan Gao,†,^‡,⊥^ Yan-Fen Niu,^†, §, ║^Fei Wang,†,^⊥^ Ping Hai,^‡,⊥^ Fang Wang, ^†, §^ Yin-Dong Fang,^⊥^ Wen-Yong Xiong,^*,†^ and Ji-Kai Liu,^*,∇, †^

^†^State Key Laboratory of Phytochemistry and Plant Resources in West China, Kunming Institute of Botany, Chinese Academy of Sciences, Kunming 650201, P. R. China

^‡^Department of Chemical Engineering, Yibin University, Yibin 644000, P. R. China

^§^Graduate University of Chinese Academy of Sciences, Beijing 100049, P. R. China

^⊥^BioBioPha Co., Ltd., Kunming 650201, P. R. China

^║^Yunnan University, Kunming 650091, P.R. China

^∇^School of Pharmaceutical Sciences, South-Central University for Nationalities, Wuhan 430074, China

The structures of compounds **1**−**5**

**Content list:**

**S1.**^1^H NMR spectrum (600 MHz,pyridine-*d*_5_) of **1**.

**S2.**^13^C NMR (DEPT) spectrum (150 MHz, pyridine-*d*_5_) of **1**.

**S3.** HMBC spectrum (600 MHz, pyridine-*d*_5_) of **1**.

**S4.** HSQC spectrum (600 MHz, pyridine-*d*_5_) of **1**.

**S5.**ROESY spectrum (600 MHz, pyridine-*d*_5_) of**1**.

**S6.**^1^H NMR spectrum (600 MHz,DMSO-*d*_6_) of **2**.

**S7.**^13^C NMR (DEPT) spectrum (150 MHz, DMSO-*d*_6_) of **2**.

**S8.** HMBC spectrum (600 MHz, DMSO-*d*_6_) of **2**.

**S9.** HSQC spectrum (600 MHz, DMSO-*d*_6_) of **2**.

**S10.**ROESY spectrum (600 MHz, DMSO-*d*_6_) of**2**.

**S11.**^1^H NMR spectrum (600 MHz,CDCl_3_) of **2**.

**S12.**^13^C NMR (DEPT) spectrum (150 MHz, CDCl_3_) of **2**.

**S13.**HMBC spectrum (600 MHz, CDCl_3_) of **2**.

**S14.**HSQC spectrum (600 MHz, CDCl_3_) of **2**.

**S15.**ROESY spectrum (600 MHz, CDCl_3_) of**2**.

**S16.**^1^H NMR spectrum (600 MHz,CDCl_3_) of **3**.

**S17.**^13^C NMR spectrum (150 MHz, CDCl_3_) of **3**.

**S18.**HMBC spectrum (600 MHz, CDCl_3_) of **3**.

**S19.**HSQC spectrum (600 MHz, CDCl_3_) of **3**.

**S20.**ROESY spectrum (600 MHz, CDCl_3_) of **3**.

**S21.**^1^H NMR spectrum (600 MHz,CDCl_3_) of **4**.

**S22.**^13^C NMR spectrum (150 MHz, CDCl_3_) of **4**.

**S23.**HMBC spectrum (600 MHz, CDCl_3_) of **4**.

**S24.**HSQC spectrum (600 MHz, CDCl_3_) of **4**.

**S25.**ROESY spectrum (600 MHz, CDCl_3_) of **4**.

**S26.**^1^H NMR spectrum (600 MHz, methanol-*d*_4_) of **5**.

**S27.**^13^C NMR (DEPT) spectrum (150 MHz, methanol-*d*_4_) of **5**.

**S28.**HMBC spectrum (600 MHz, methanol-*d*_4_) of **5**.

**S29.**HSQC spectrum (600 MHz, methanol-*d*_4_) of **5**.

**S30.**ROESY spectrum (600 MHz, methanol-*d*_4_) of **5**.

**S1.**^1^H NMR spectrum (600 MHz, pyridine-*d*_5_) of**1**.
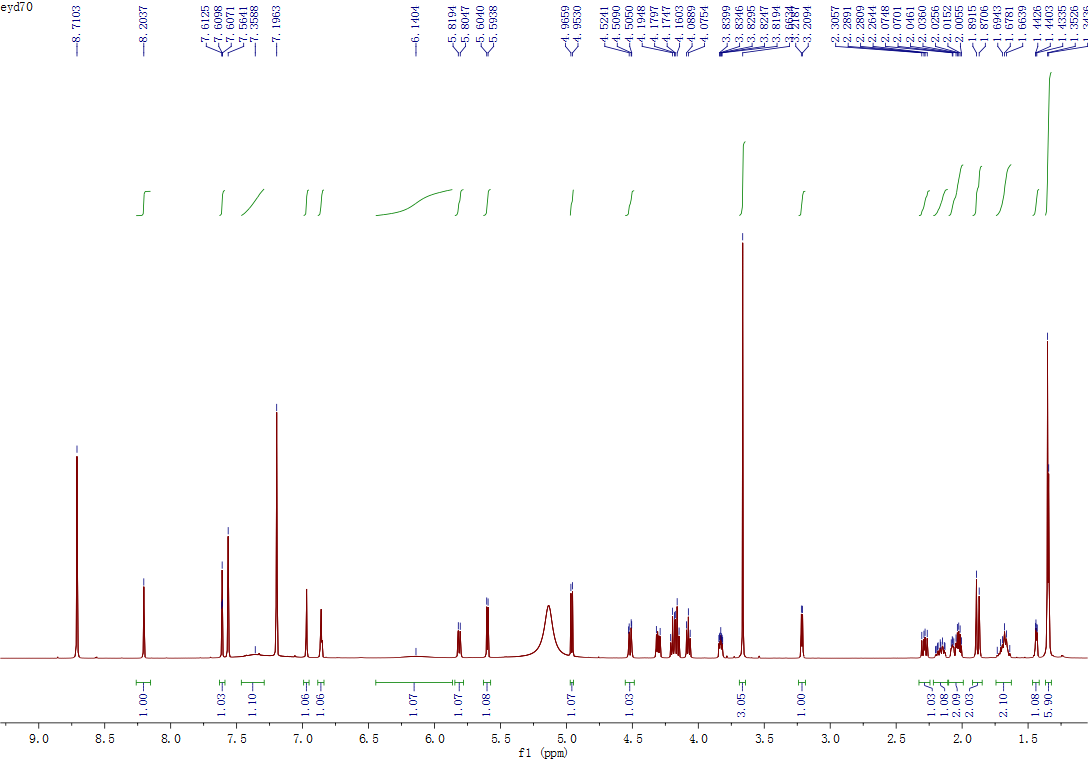


**S2.**^13^C NMR (DEPT) spectrum (150 MHz, pyridine-*d*_5_) of **1**.
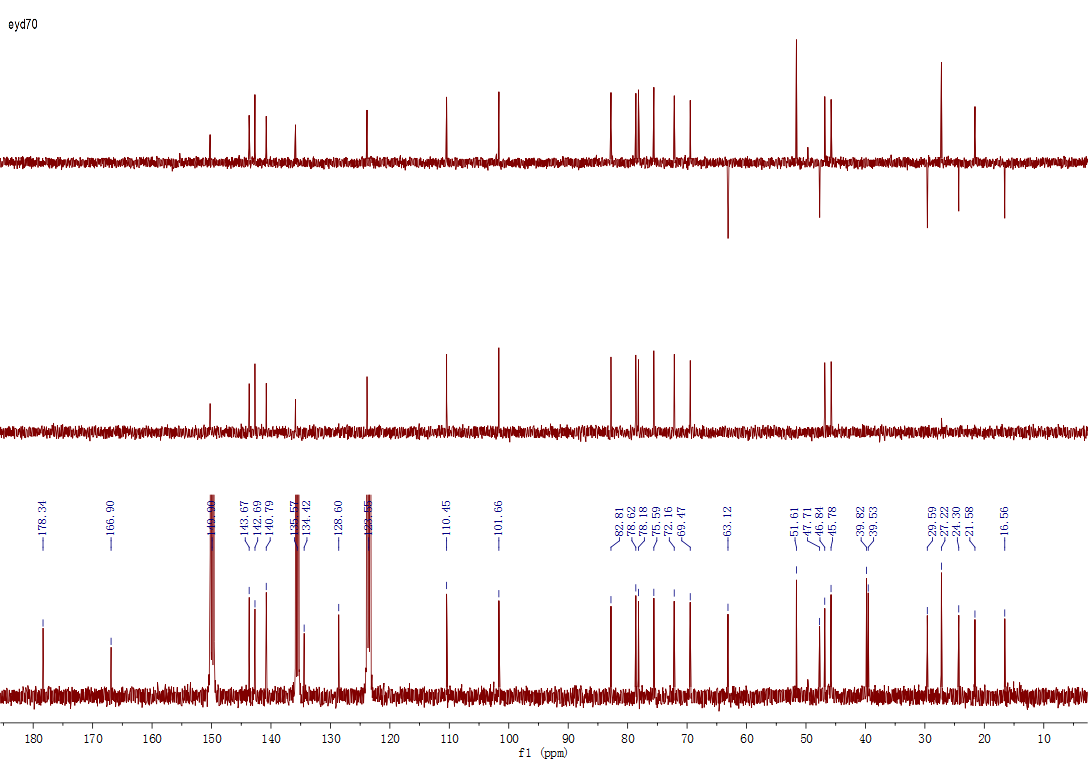


**S3.** HMBC spectrum (600 MHz, pyridine-*d*_5_) of **1**.
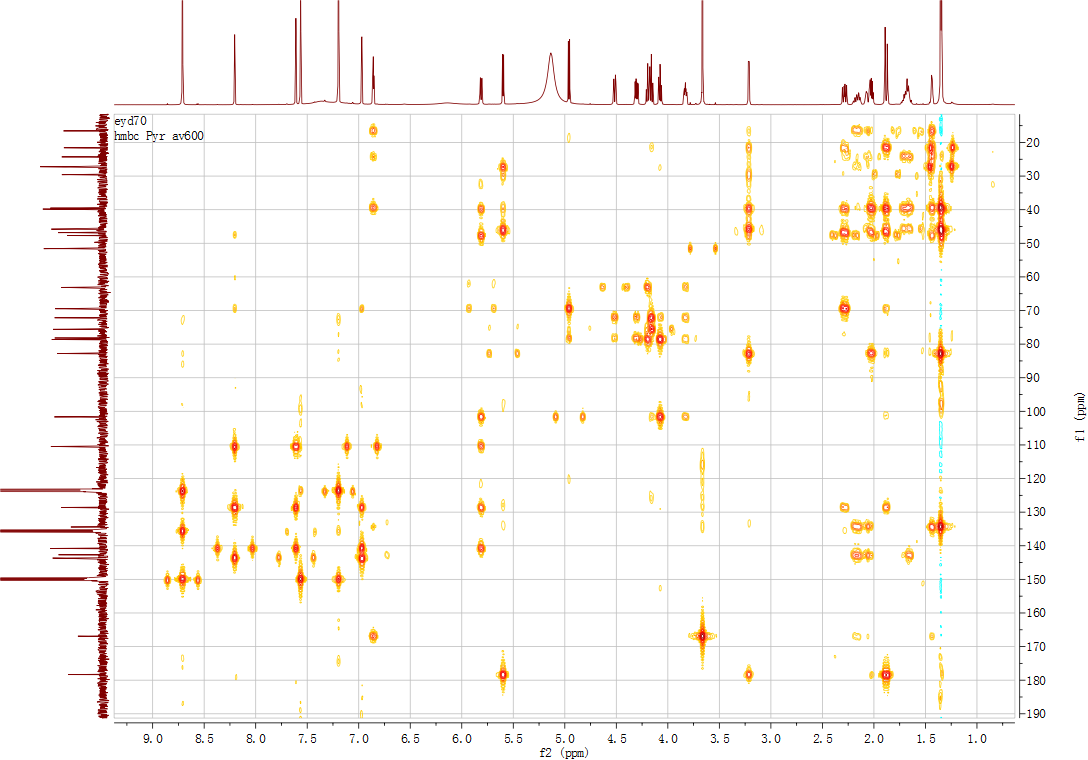


**S4.** HSQC spectrum (600 MHz, pyridine-*d*_5_) of **1**.
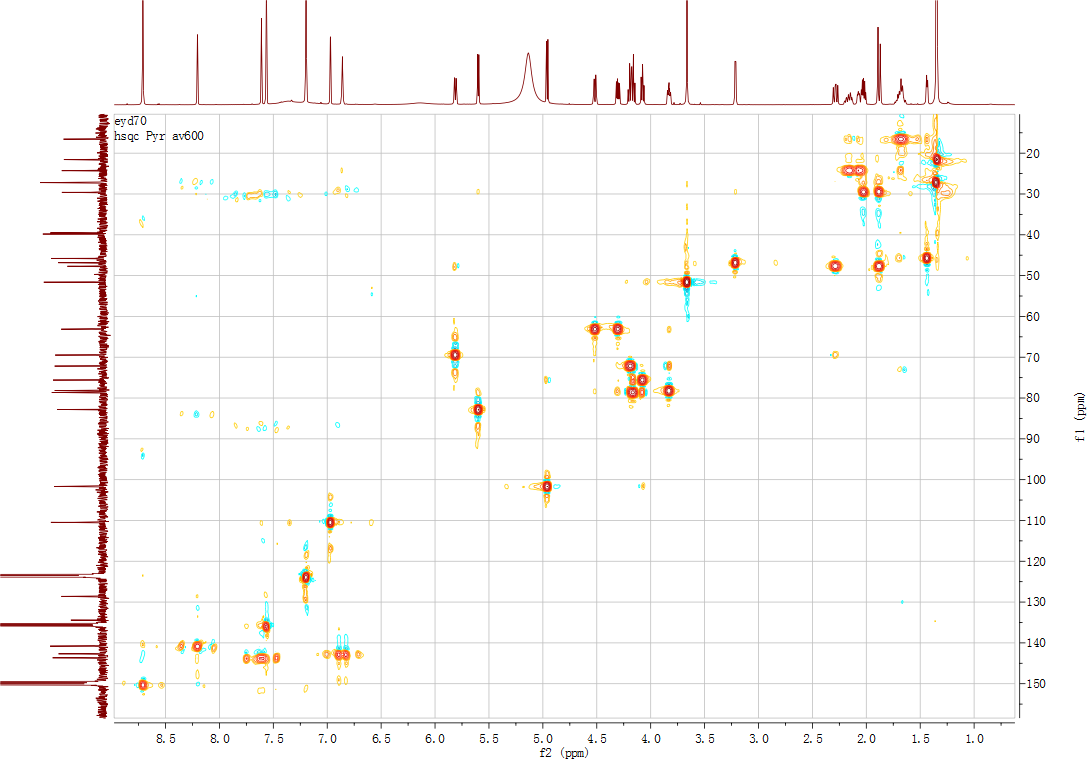


**S5.**ROESY spectrum (600 MHz, pyridine-*d*_5_) of **1**.
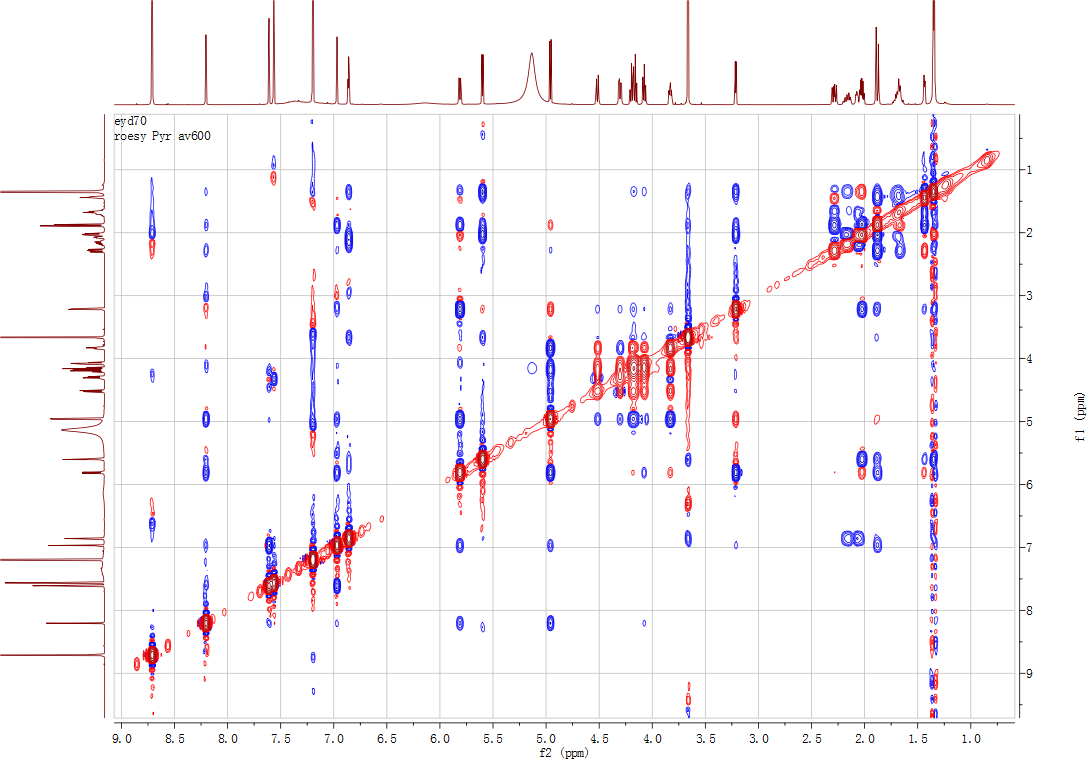


**S6.**^1^H NMR spectrum (600 MHz, DMSO-*d*_6_) of **2**.
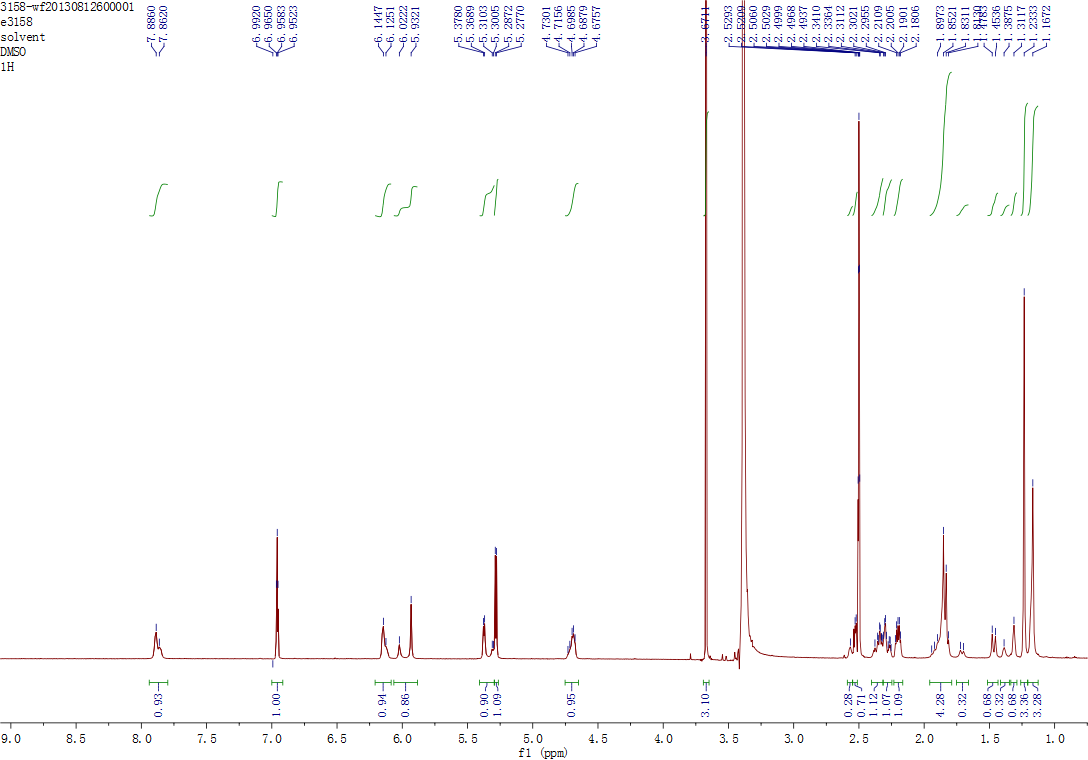


**S7.**^13^C NMR (DEPT) spectrum (150 MHz, DMSO-*d*_6_) of **2**.
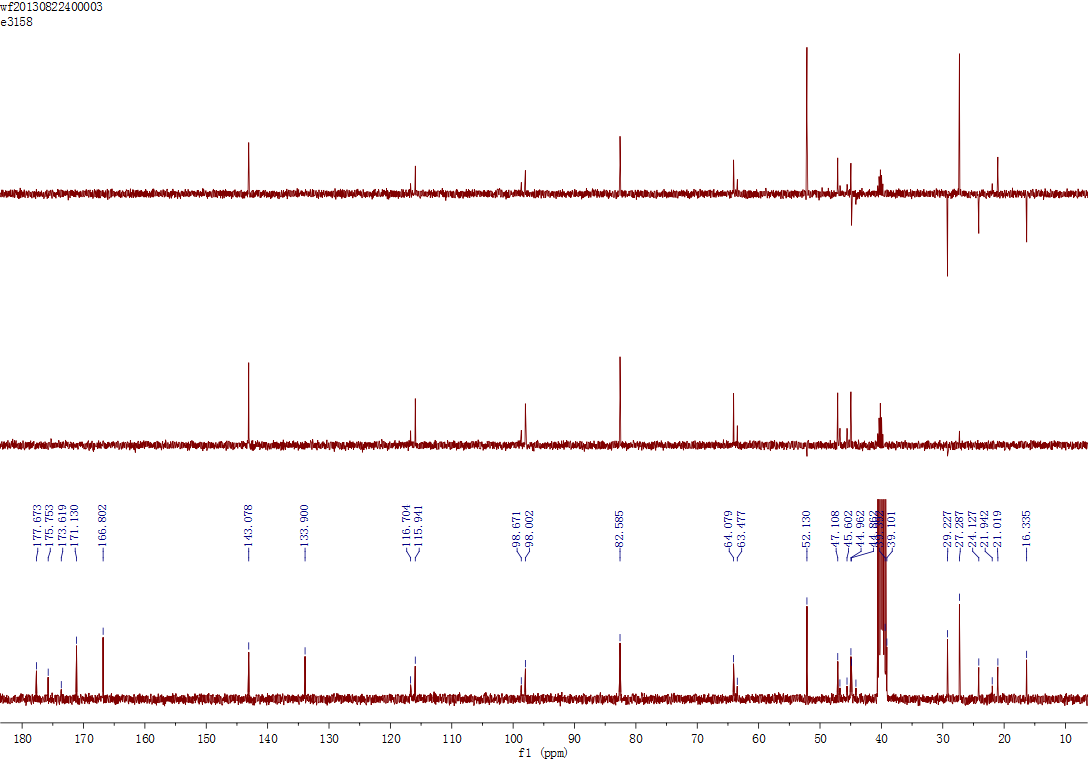


**S8.**HMBC spectrum (600 MHz, DMSO-*d*_6_) of **2**.
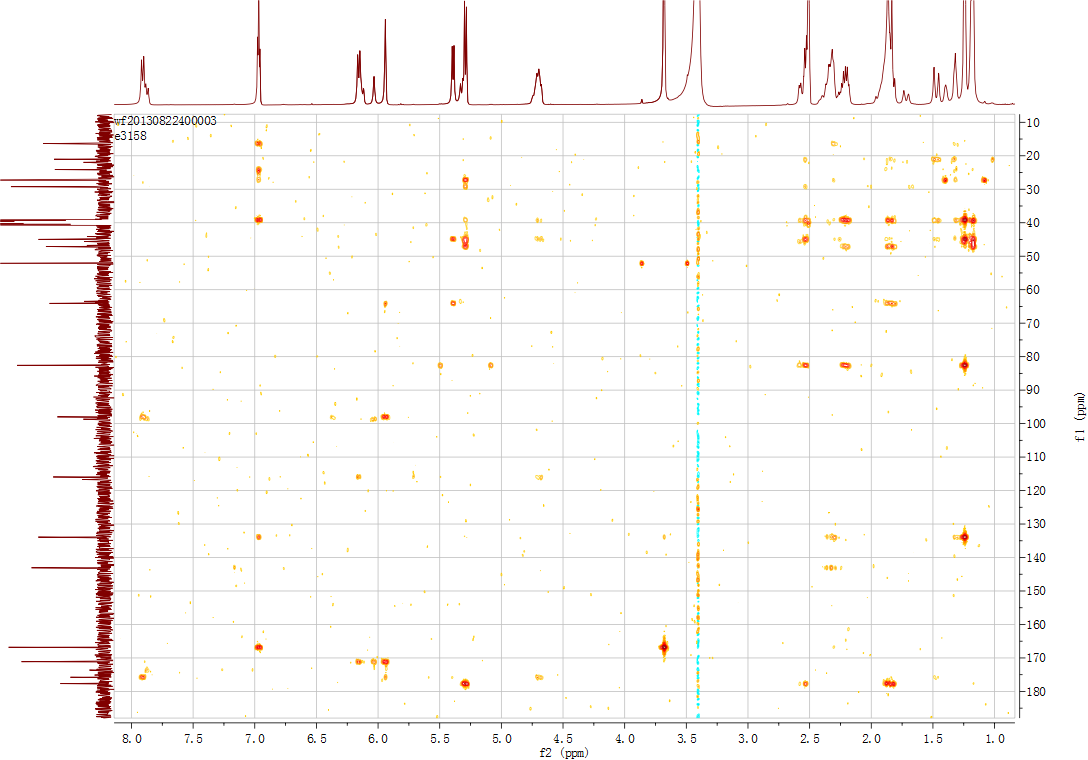


**S9.**HSQC spectrum (600 MHz, DMSO-*d*_6_) of **2**.
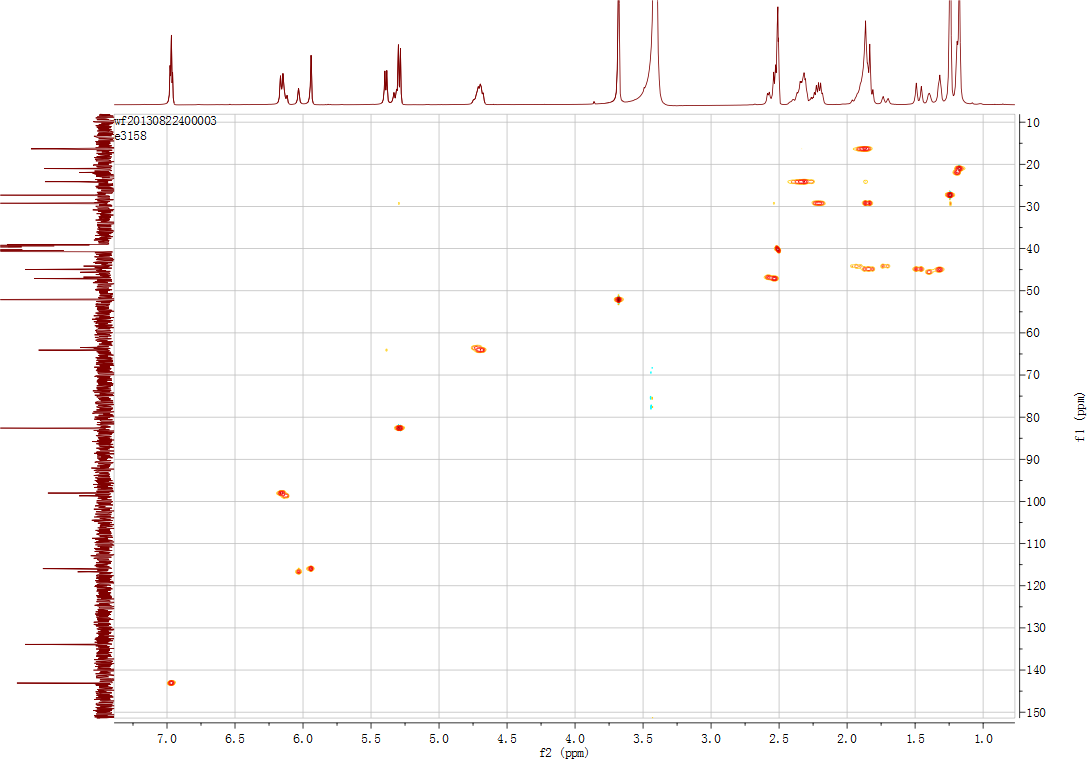


**S10.**ROESY spectrum (600 MHz, DMSO-*d*_6_) of **2**.
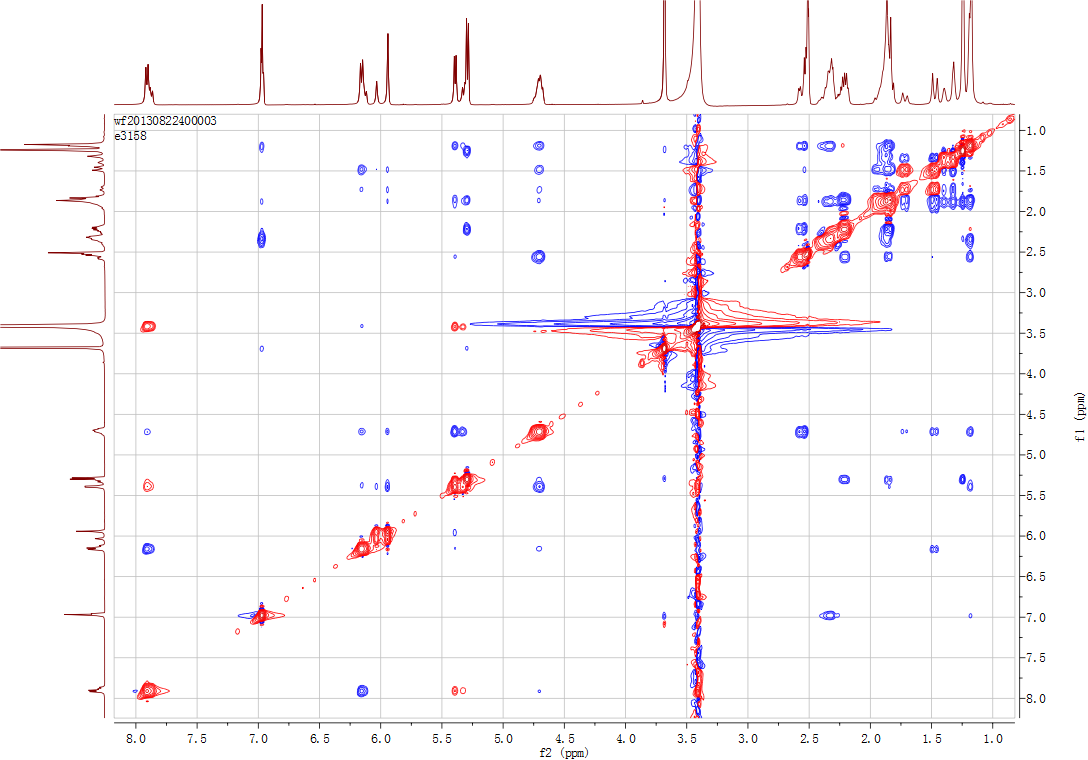


**S11.**^1^H NMR spectrum (600 MHz, CDCl_3_) of **2**.
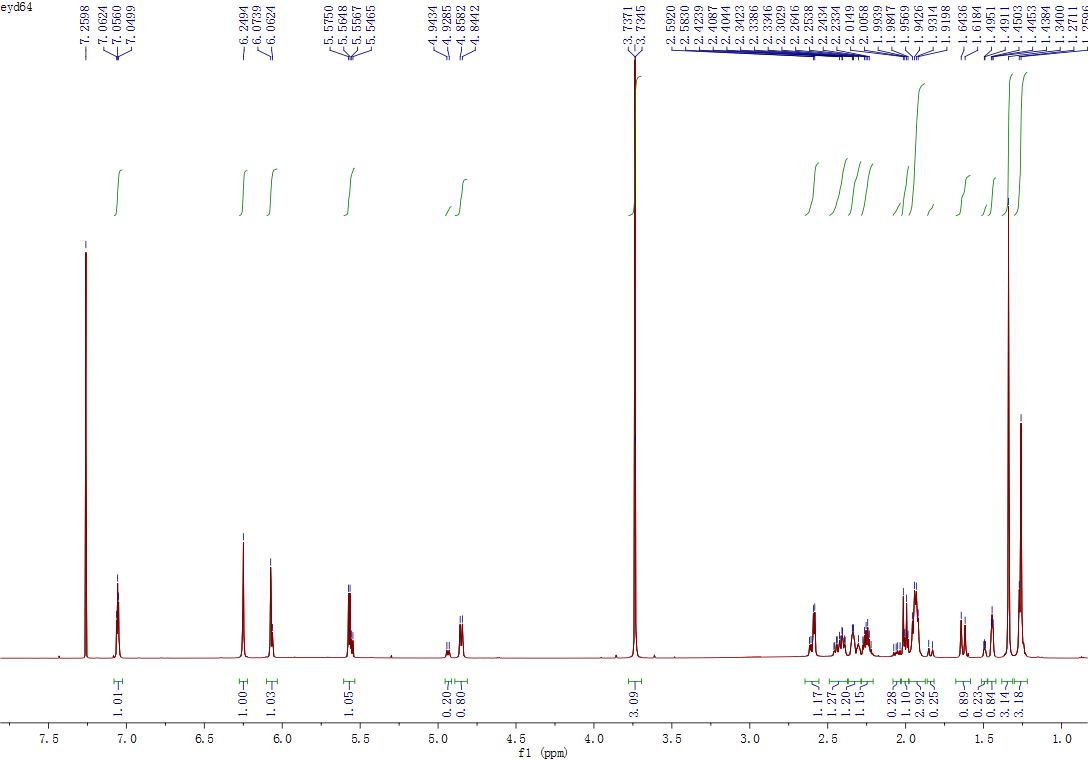


**S12.**^13^C NMR (DEPT) spectrum (150 MHz, CDCl_3_) of **2**.
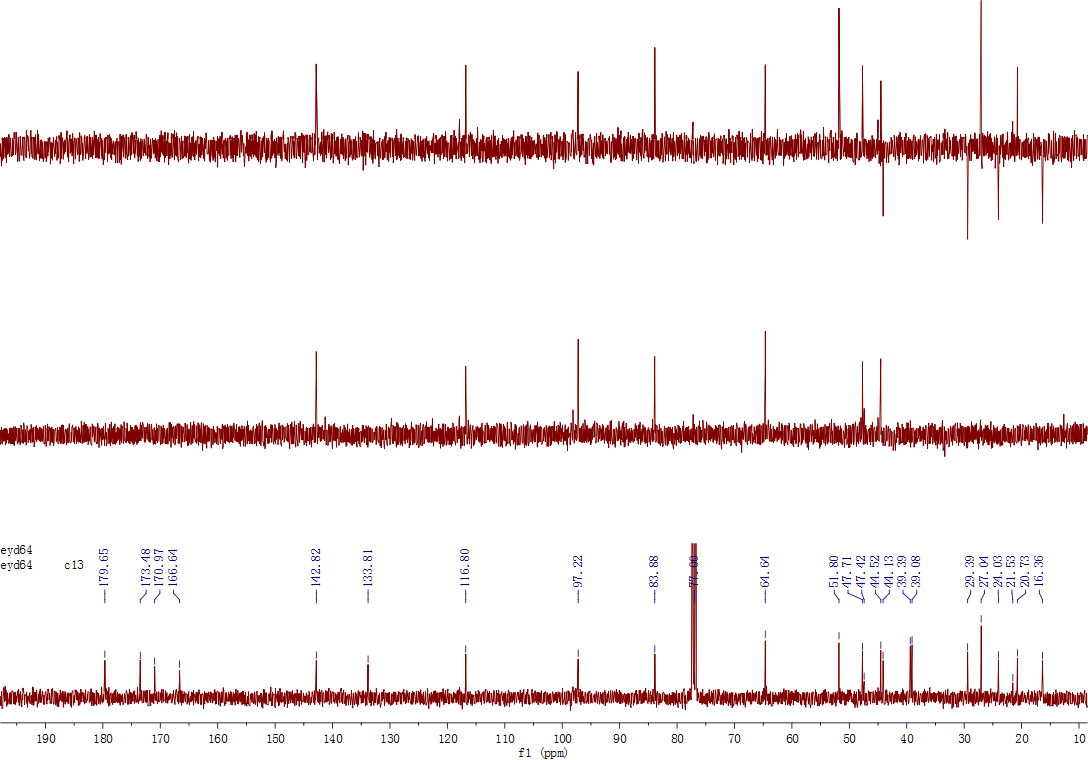


**S13.**HMBC spectrum (600 MHz, CDCl_3_) of **2**.
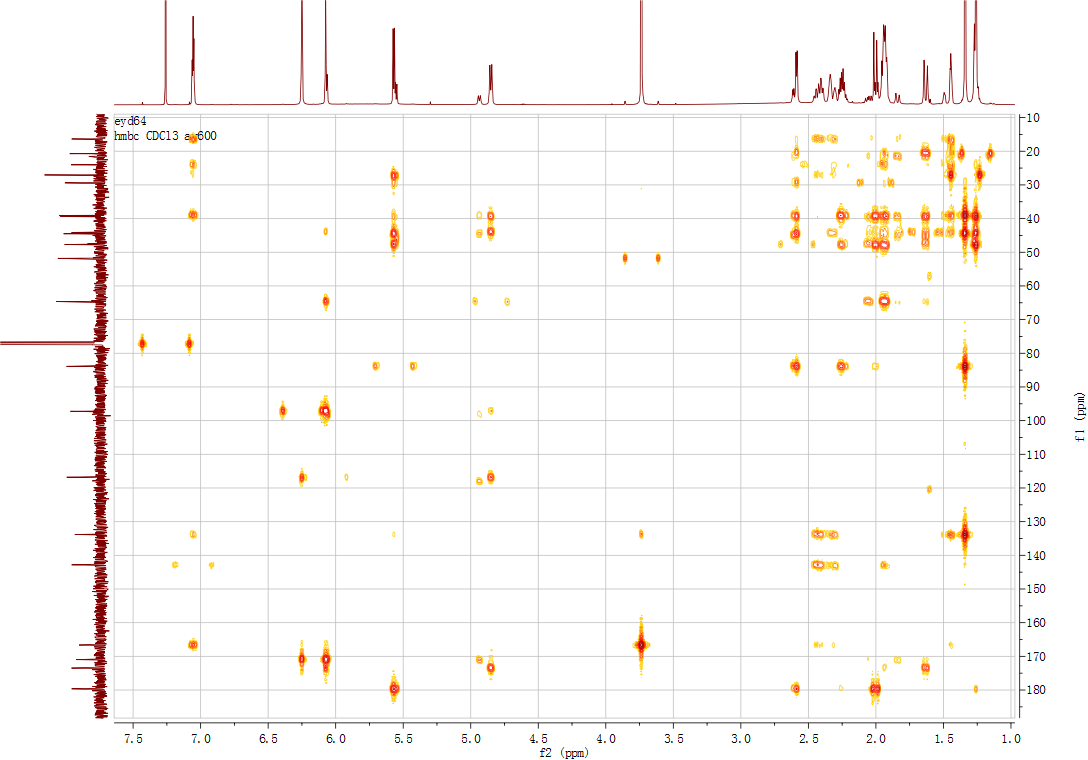


**S14.**HSQC spectrum (600 MHz, CDCl_3_) of **2**.
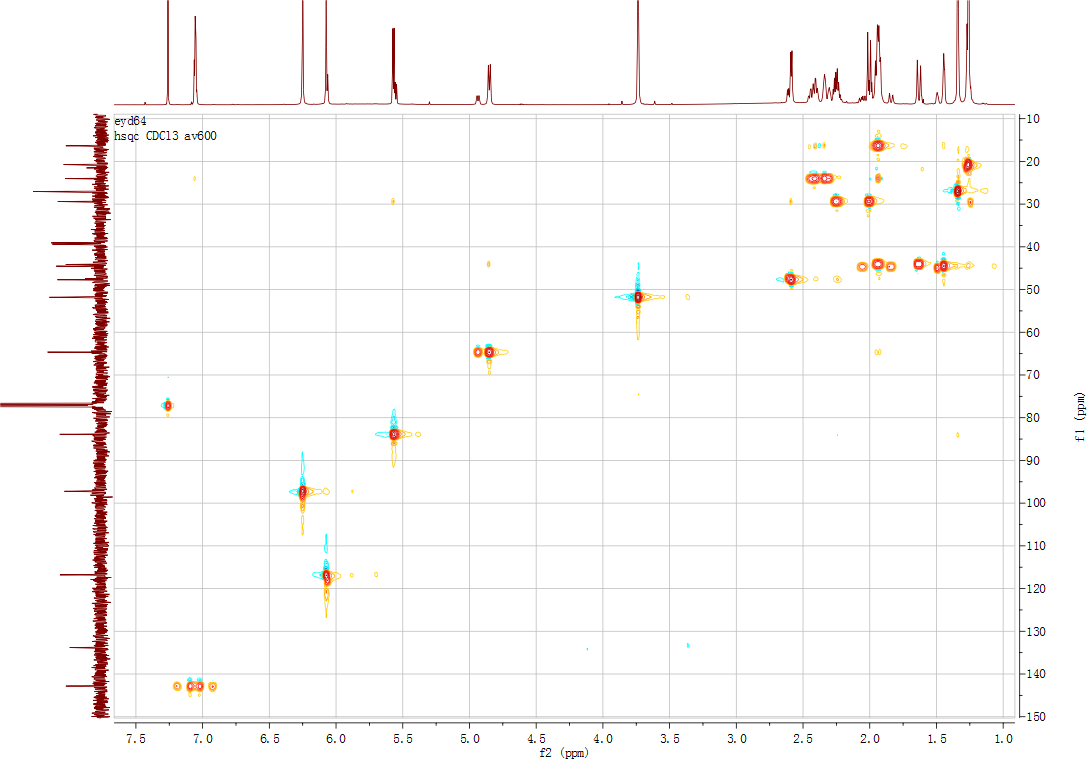


**S15.**ROESY spectrum (600 MHz, CDCl_3_) of **2**.
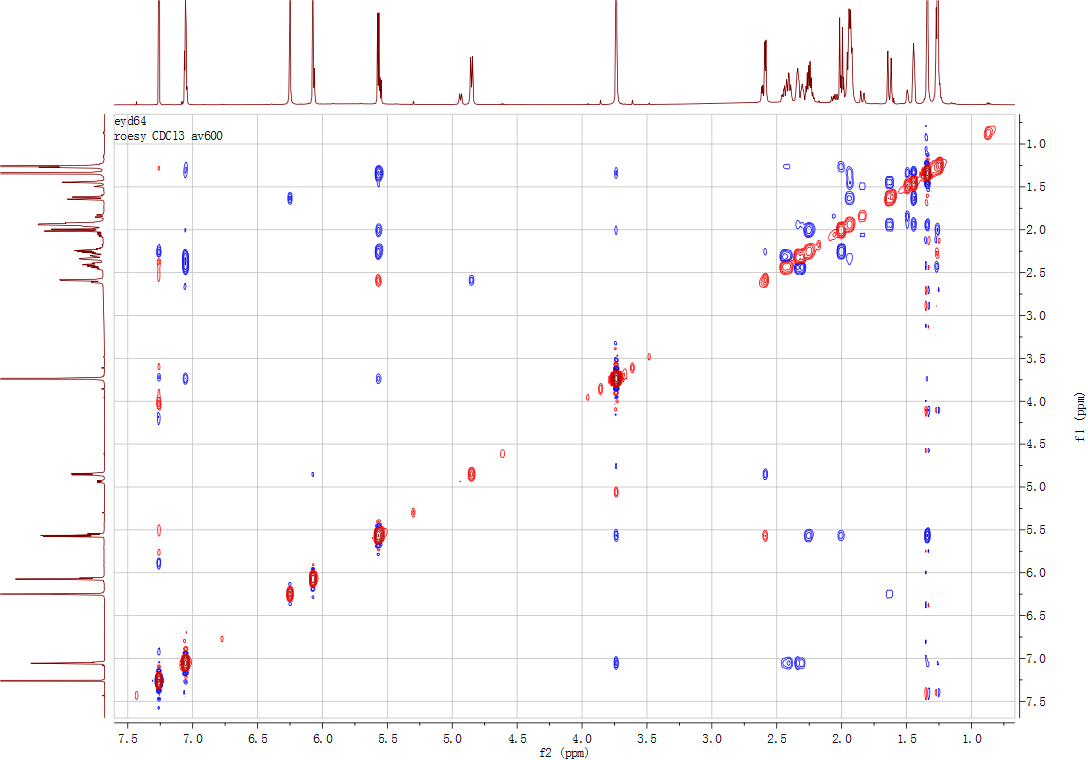


**S16.**^1^H NMR spectrum (600 MHz, CDCl_3_) of **3**.
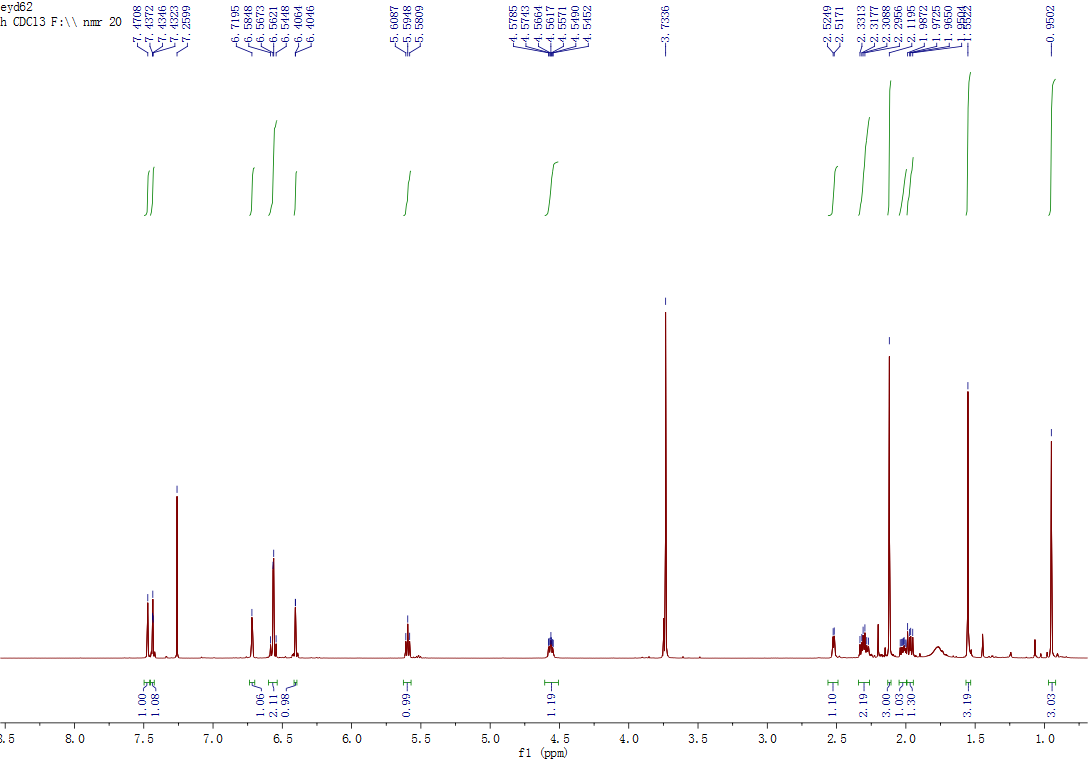


**S17.**^13^C NMR spectrum (150 MHz, CDCl_3_) of **3**.
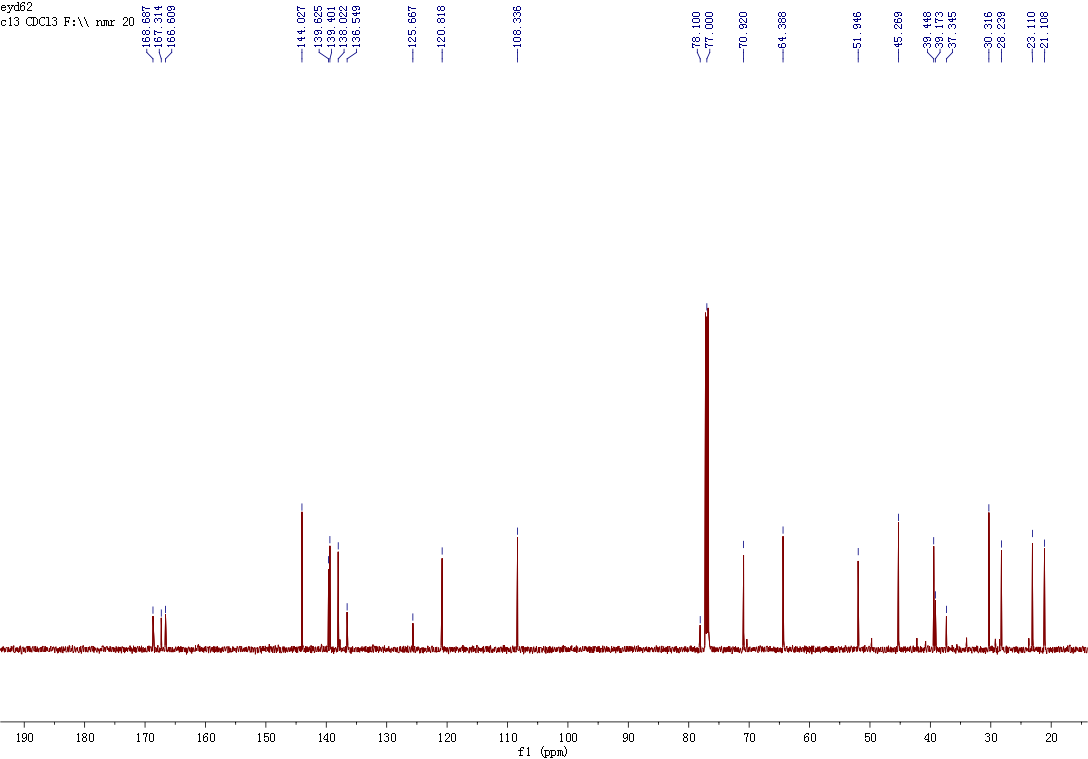


**S18.**HMBC spectrum (600 MHz, CDCl_3_) of **3**.
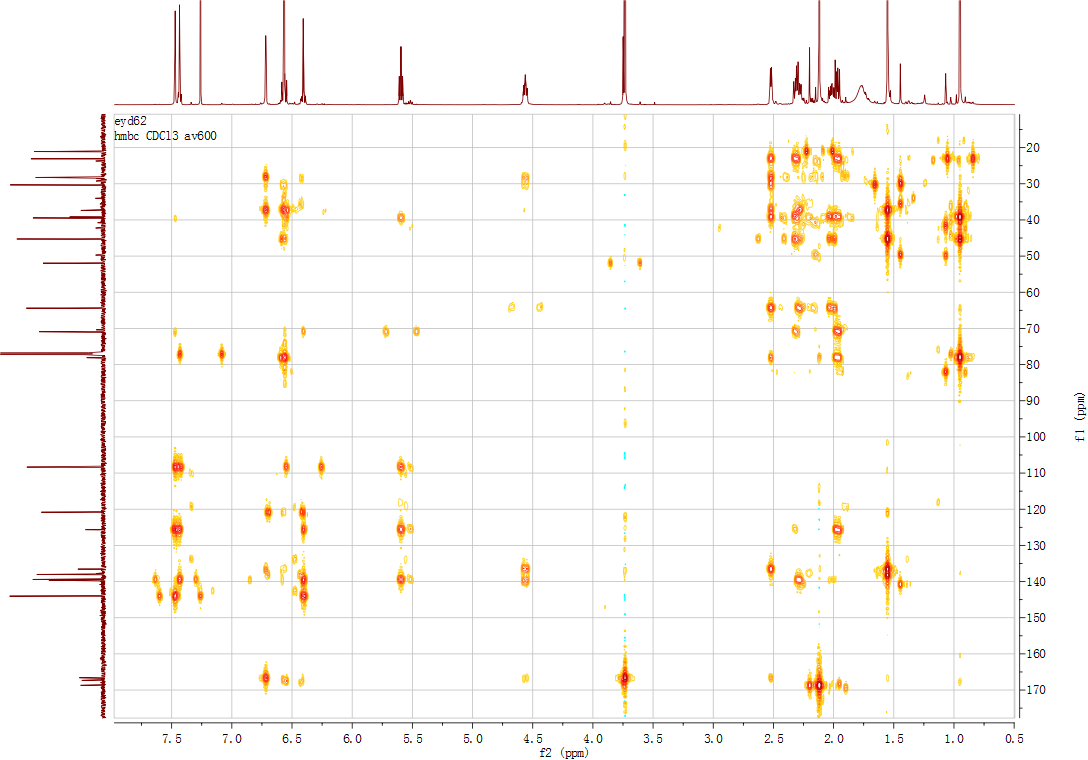


**S19.**HSQC spectrum (600 MHz, CDCl_3_) of **3**.
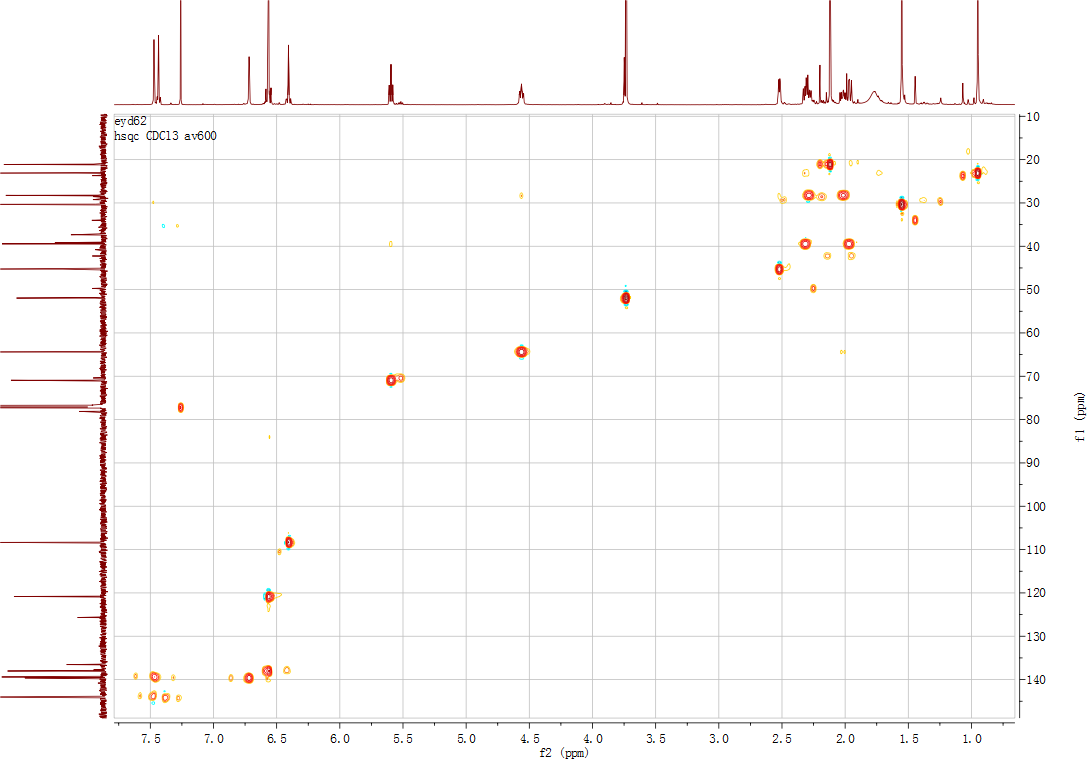


**S20.**ROESY spectrum (600 MHz, CDCl_3_) of **3**.
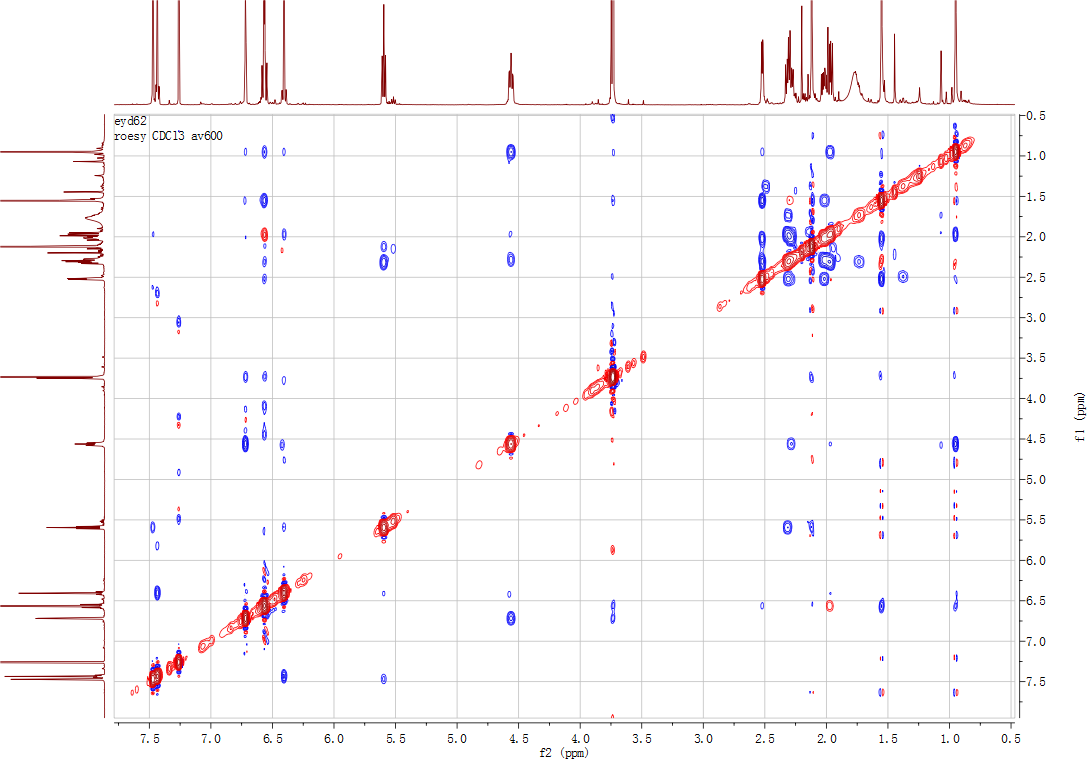


**S21.**^1^H NMR spectrum (600 MHz,CDCl_3_) of **4**.
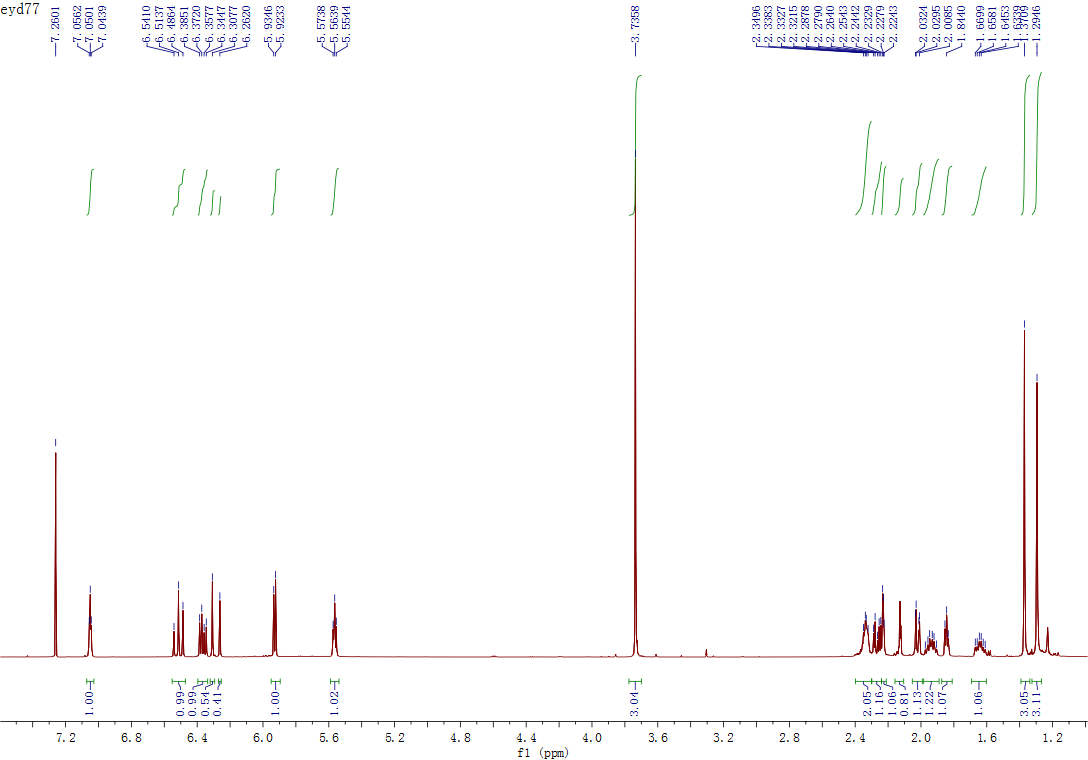


**S22.**^13^C NMR spectrum (150 MHz, CDCl_3_) of **4**.
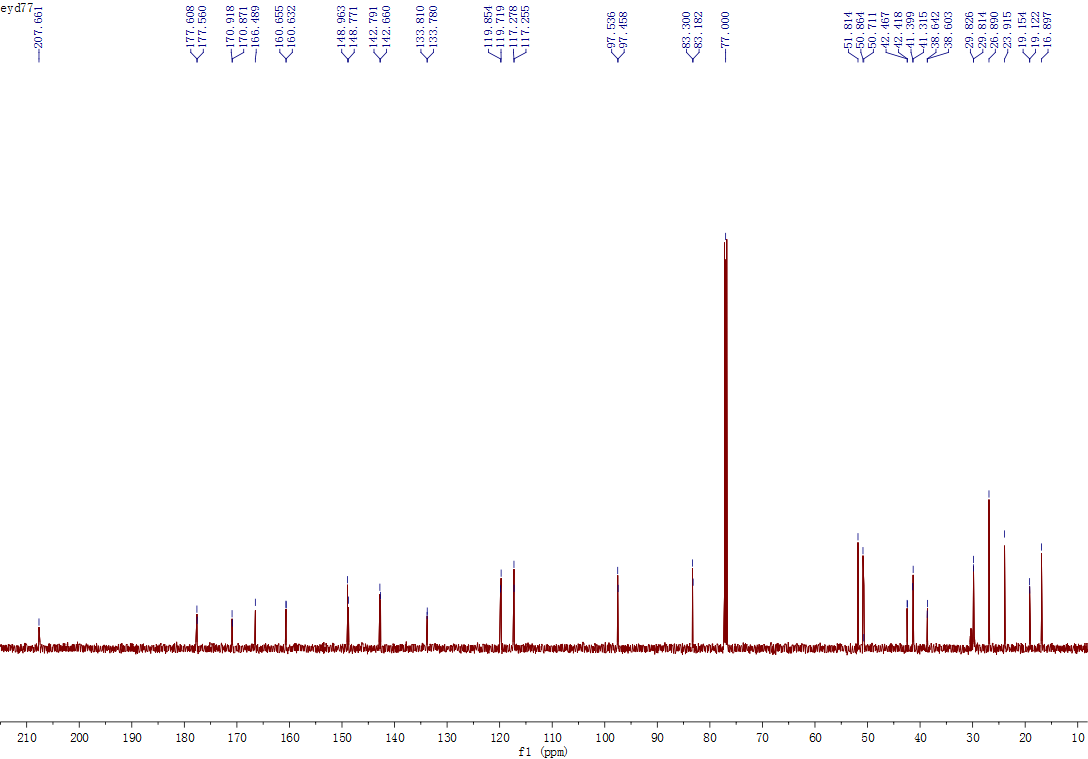


**S23.**HMBC spectrum (600 MHz, CDCl_3_) of **4**.
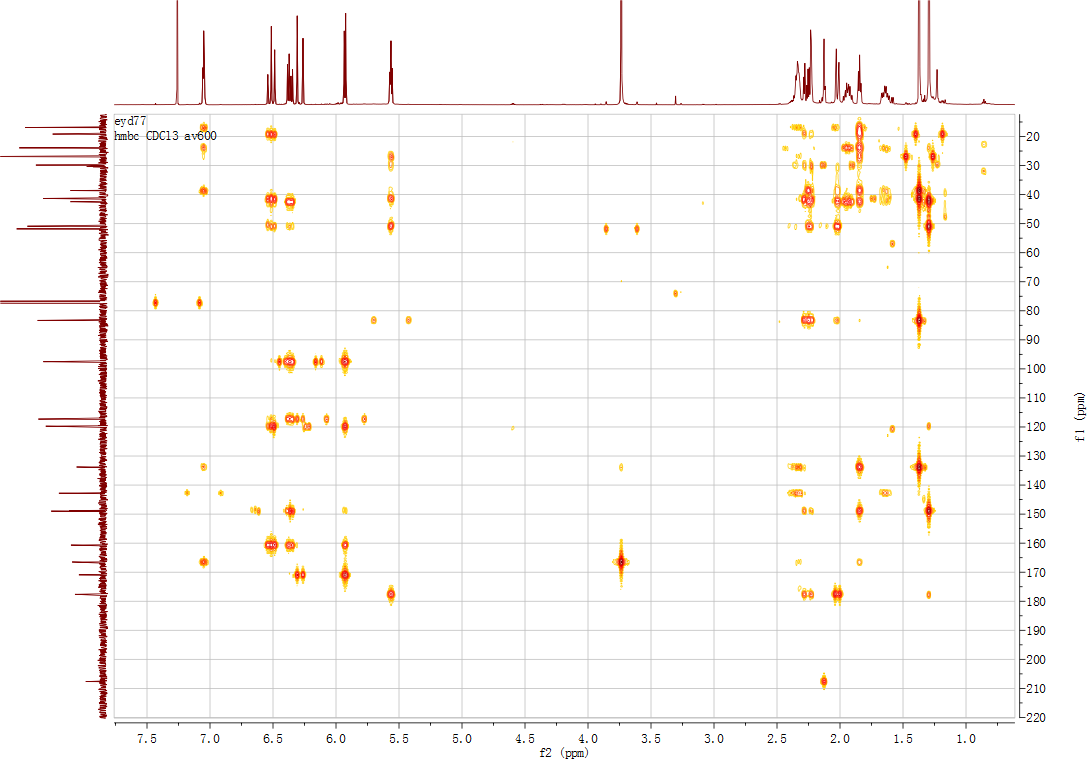


**S24.**HSQC spectrum (600 MHz, CDCl_3_) of **4**.
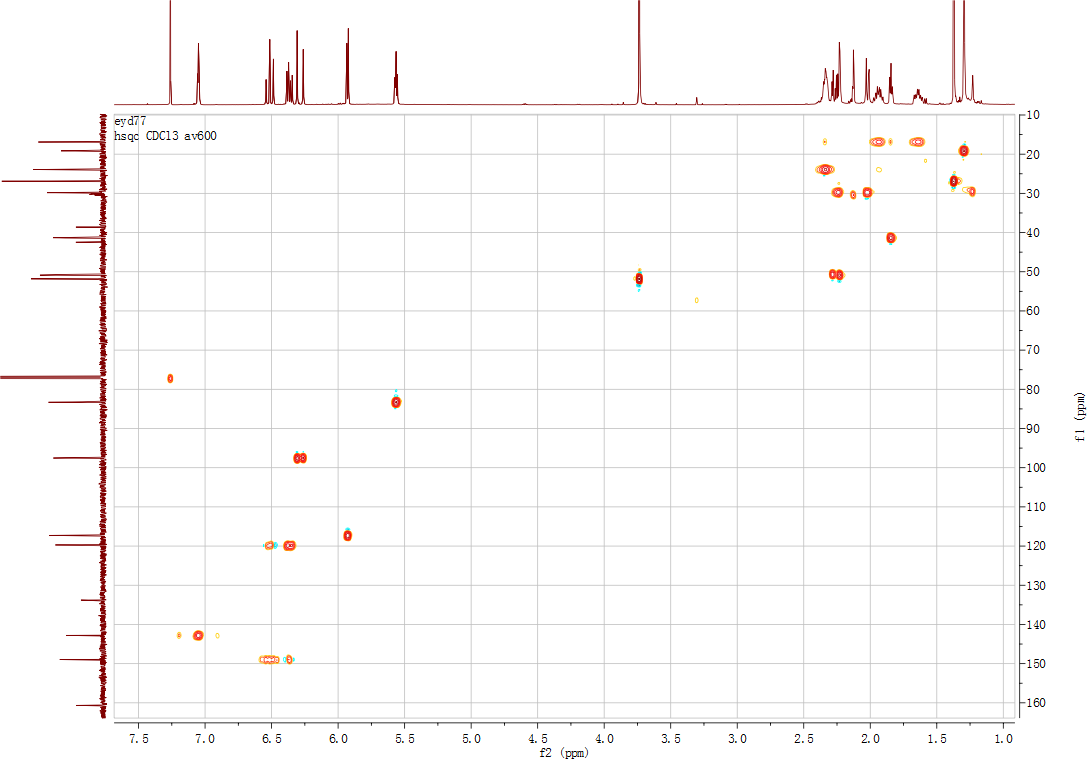


**S25.**ROESY spectrum (600 MHz, CDCl_3_) of **4**.
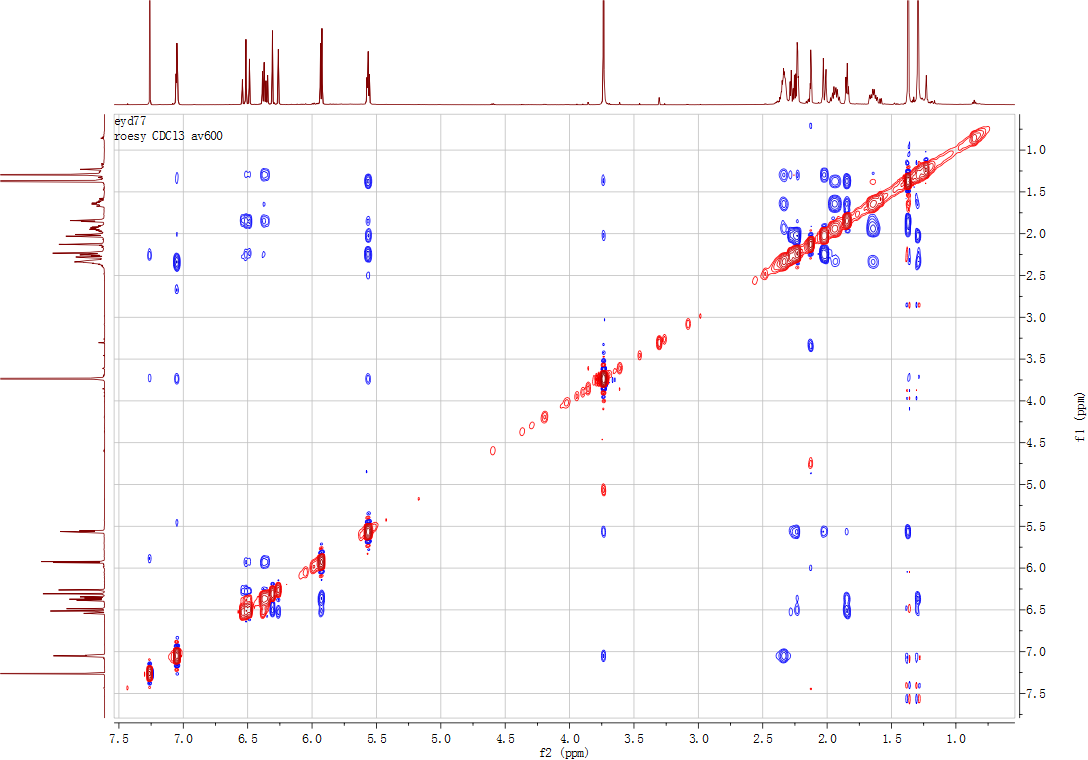


**S26.**^1^H NMR spectrum (600 MHz, methanol-*d*_4_) of **5**.
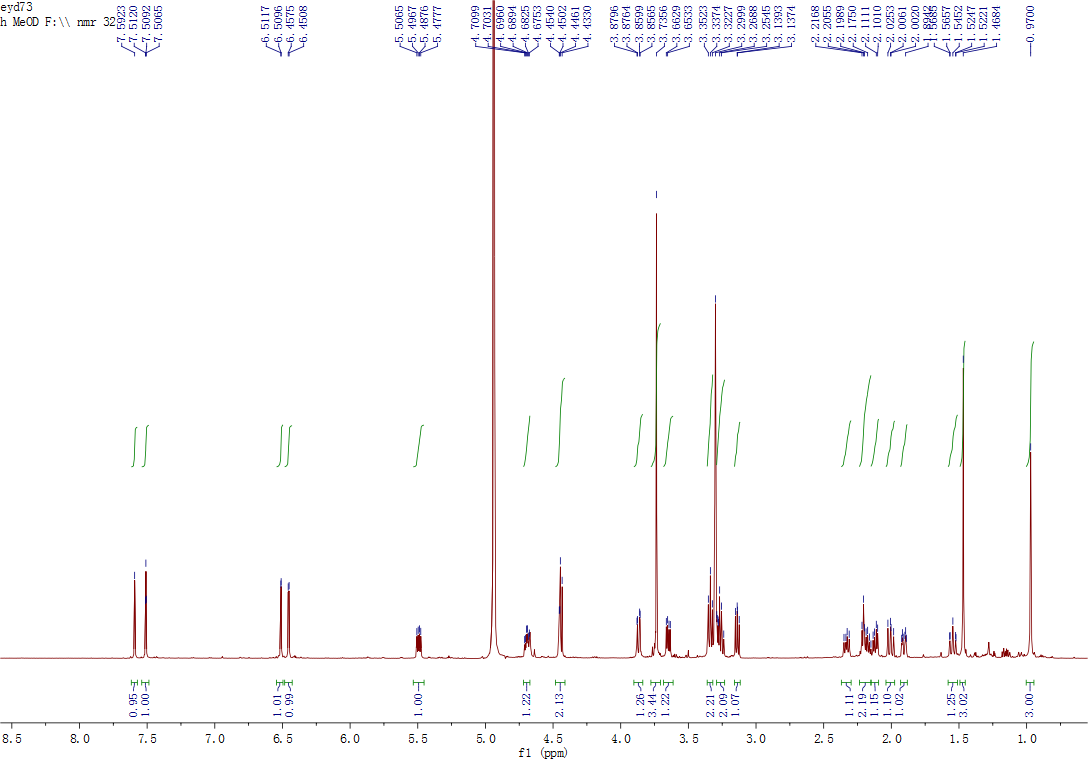


**S27.**^13^C NMR (DEPT) spectrum (150 MHz, methanol-*d*_4_) of **5**.
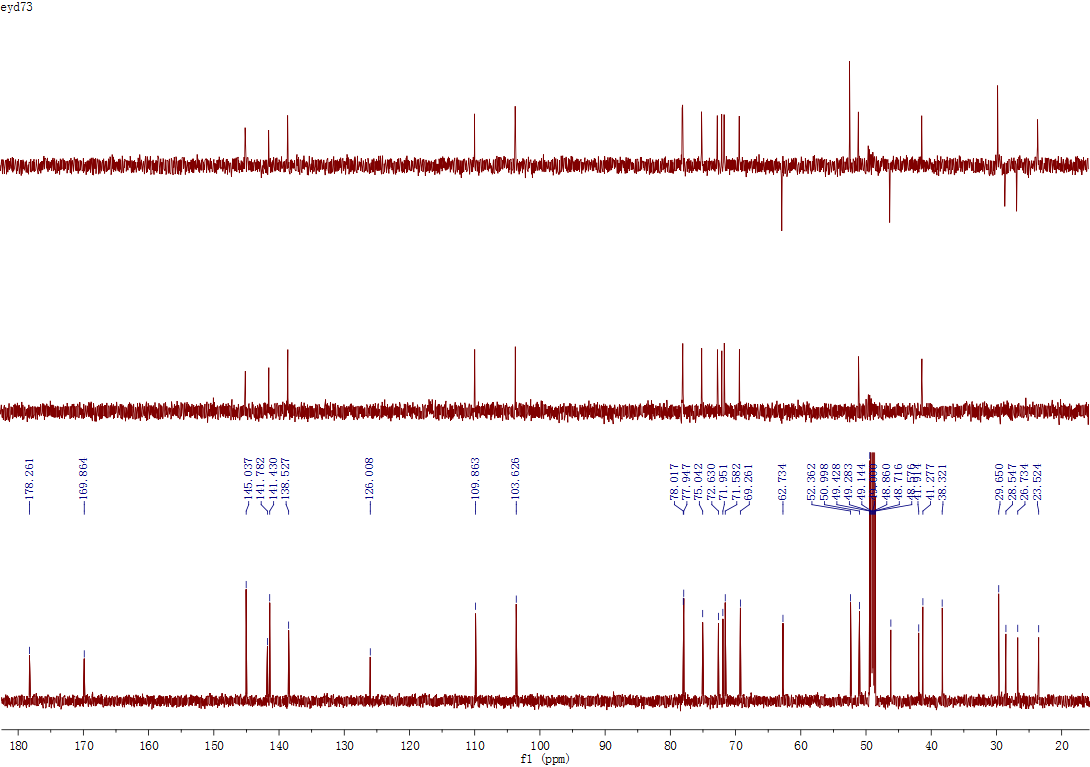


**S28.**HMBC spectrum (600 MHz, methanol-*d*_4_) of **5**.
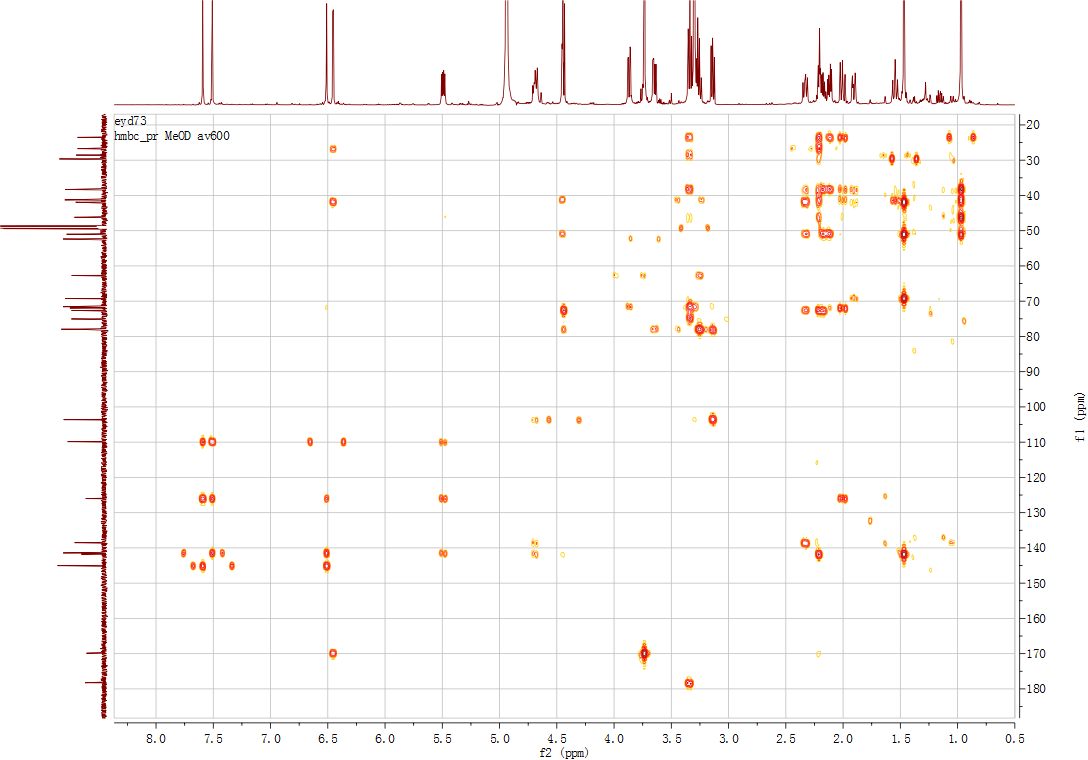


**S29.**HSQC spectrum (600 MHz, methanol-*d*_4_) of **5**.
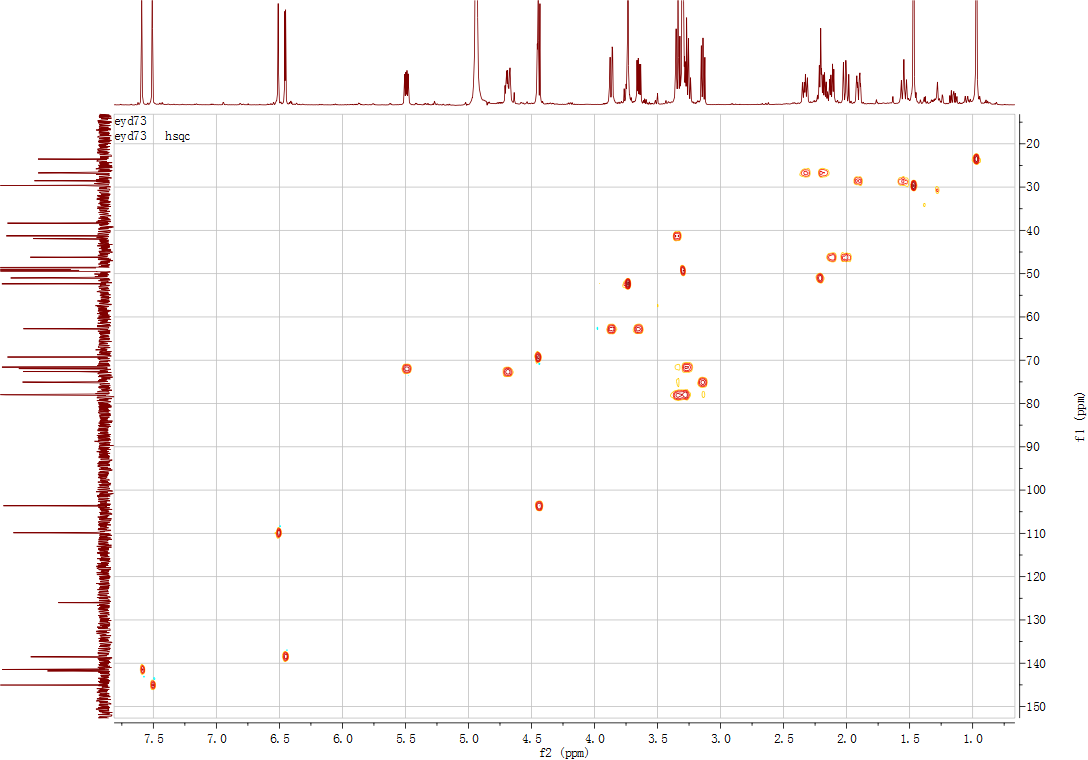


**S30.**ROESY spectrum (600 MHz, methanol-*d*_4_) of **5**.
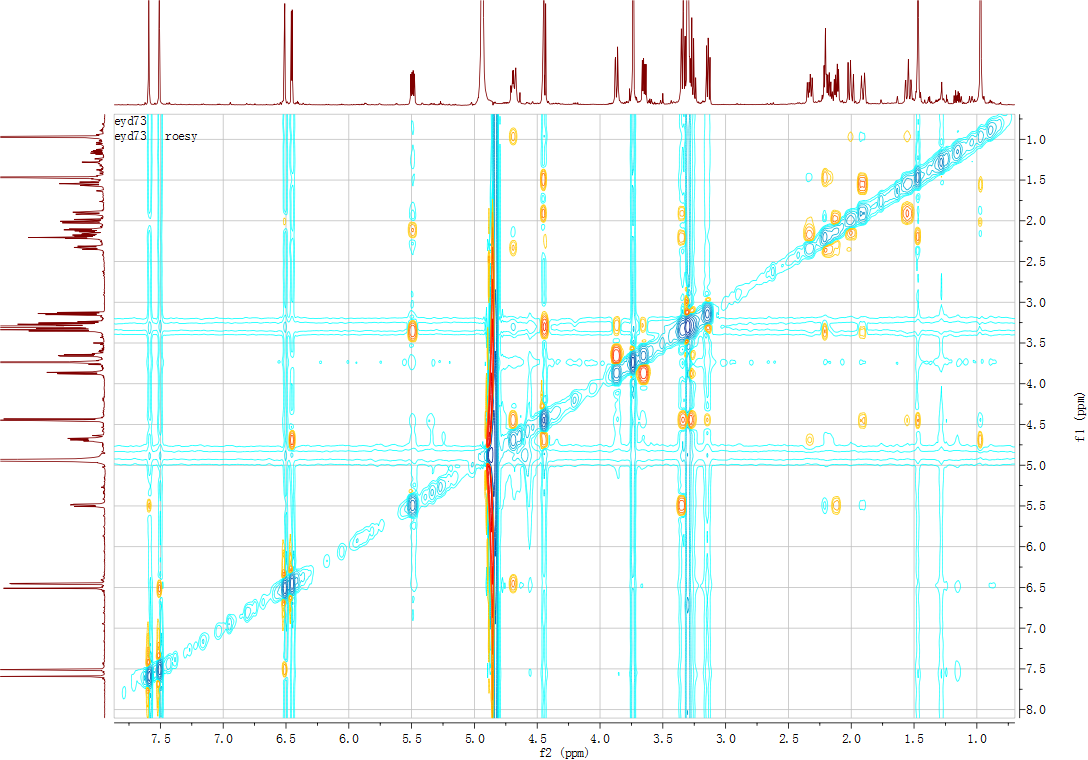

Supplement: Supplementary file 1 — Supplementary material 1 (DOCX 3118 kb) [file 13659_2016_109_MOESM1_ESM.docx]
